# Characteristics, distribution, and origin of ferruginous deposits within the Late Ordovician glaciogenic setting of Arabia

**DOI:** 10.1038/s41598-023-45563-9

**Published:** 2023-10-27

**Authors:** Abdullah Alqubalee, Anas Muhammad Salisu, Abdulwahab Muhammad Bello, Abdulkarim Al-Hussaini, Khalid Al-Ramadan

**Affiliations:** 1 Center for Integrative Petroleum Research, College of Petroleum Engineering and Geosciences, King Fahd University of Petroleum and Minerals, 31261 Dhahran, Saudi Arabia; 2 Geosciences Department, College of Petroleum Engineering and Geosciences, King Fahd University of Petroleum and Minerals, 31261 Dhahran, Saudi Arabia; 3 Exploration Organization, Saudi Aramco, 31311 Dhahran, Saudi Arabia

**Keywords:** Sedimentology, Stratigraphy

## Abstract

Ferruginous deposits are iron-rich sediments or sedimentary rocks found in various sizes, shapes, and compositions within sedimentary strata in different depositional settings. This study investigates the characteristics, distribution, and origin of ferruginous deposits found in the Late Ordovician glaciogenic Sarah Formation and surrounding deposits in central Saudi Arabia. Several types of ferruginous deposits have been identified through field observations and laboratory investigations, including thin-section petrography, geochemical, surface, and bulk mineralogical analyses, and computed tomography scans. The identified ferruginous deposits include solid and rinded concretions, pipes, layers, ferricretes, liesegang bands, and fracture infills. They were associated with the periglacial and proglacial facies of the Sarah Formation. For instance, ferruginous deformed layers were mainly observed in subglacial facies, while rinded concretions occurred in bleached glaciofluvial facies. Ferruginous deposits were also found in the uppermost parts of non-glacial facies, such as the shallow marine Quwarah Member of the Qasim Formation and the braided deltaic Sajir Member of the Saq Formation. Compositionally, goethite was the dominant iron oxide mineral in all ferruginous deposits, and it is mostly distributed as cement, filling pore spaces. In comparison to ferruginous deposits reported in different depositional settings on Earth and Mars, the studied ferruginous deposits in an ancient glaciogenic setting exhibit different mineralogical characteristics. Specifically, the studied solid concretions are less abundant and primarily amalgamated, while the rinded concretions appear to be more mature than those reported in other depositional environments. This study suggests that the weathered basement rocks of the Arabian Shield were the primary source of iron. The iron-bearing rocks were eroded and transported by Hirnantian glaciation and deglaciation processes.

## Introduction

Ferruginous deposits, defined as deposits with high concentrations of iron oxides, are widely reported to occur in several geological formations worldwide, consisting of several morphologies and sizes, i.e., spherical and discoidal rinded concretions, ferricrete, liesegang bandings, pipes, columns, banded iron formation, and ooidal ironstone^1–16^. Over the last decades, many studies have used the ferruginous deposits for various investigations in planetary science^1,2^, reservoir and aquifer studies^17^, and many other applications^18^. Despite the growing interest in establishing the characteristics of ferruginous deposits and their origin, the majority of published studies are limited to analyzing ferruginous concretions developed in fluvial-to-aeolian depositional settings, e.g., the Jurassic aeolian Navajo sandstones of the United States^1,2,9,19^, the Pleistocene fluvial pre-Riss Formation in the Netherlands^11,20^, and the Cretaceous Djadochta Formation in southern Mongolia^21^. This has resulted in some ambiguity in the literature regarding whether these ferruginous concretions would have different characteristics when they form in sandstones of different origins than aeolian and fluvial settings.

Iron oxides, including hydroxide and oxide-hydroxides minerals, in sandstones are evident by color changes that depend on the bleaching state^2,9,22^ and indicate chemical interactions with host rocks. "Unbleached" (or reddish, brownish) sandstones contain an amount of iron oxide, while "bleached" (or whitish, yellowish) are considered iron oxides depleted. Both physical and chemical changes observed in the ferruginous deposits led several researchers to hypothesize the origin of iron oxide concretions, liesegang bandings, pipes, and columns^9,10,22^. Other ferruginous types, i.e., ferricretes, might be generally linked to the permeability variation of the host rocks^1^.

In central Saudi Arabia, the Early Paleozoic glaciogenic and shallow marine sandstones contain ferruginous deposits with variable abundances, sizes, geometries, and compositions^23^. Such variability might reflect various mechanisms that led to the formation and distribution of iron oxides in such strata. This study investigates the characteristics and distribution patterns of the ferruginous deposits within the Early Paleozoic glaciogenic and shallow marine sandstones. It also discusses the potential sources of iron in the studied area. In addition, this study qualitatively compares the studied ferruginous deposits with some of the well-known studied ferruginous deposits on Earth (i.e., the Jurassic Navajo ferruginous deposits) and Mars (i.e., blueberries) to highlight the similarities and differences. It is expected to provide some insights into the formational mechanism of such deposits to understand their evolution through geological time.

## Geological settings and stratigraphy

During the Paleozoic Era, Arabia was attached to Africa as part of the Gondwana supercontinent^24^ and mainly represented a wide passive margin^25^. Continental to shallow marine siliciclastic deposits dominated most of the Arabian Plate following the Najd Rift Phase^26^, representing four full megasequences of the Arabian Plate (AP), AP2–AP5^27,28^. During this Era, two glaciation events occurred, including the Late Ordovician (Hirnantian)^29^ and the Late Carboniferous–Early Permian (Latest Visean–Latest Sakmarian) events. In Saudi Arabia, the Paleozoic deposits mainly outcrop in central, northwest, and southwestern regions.

**Figure 1:**
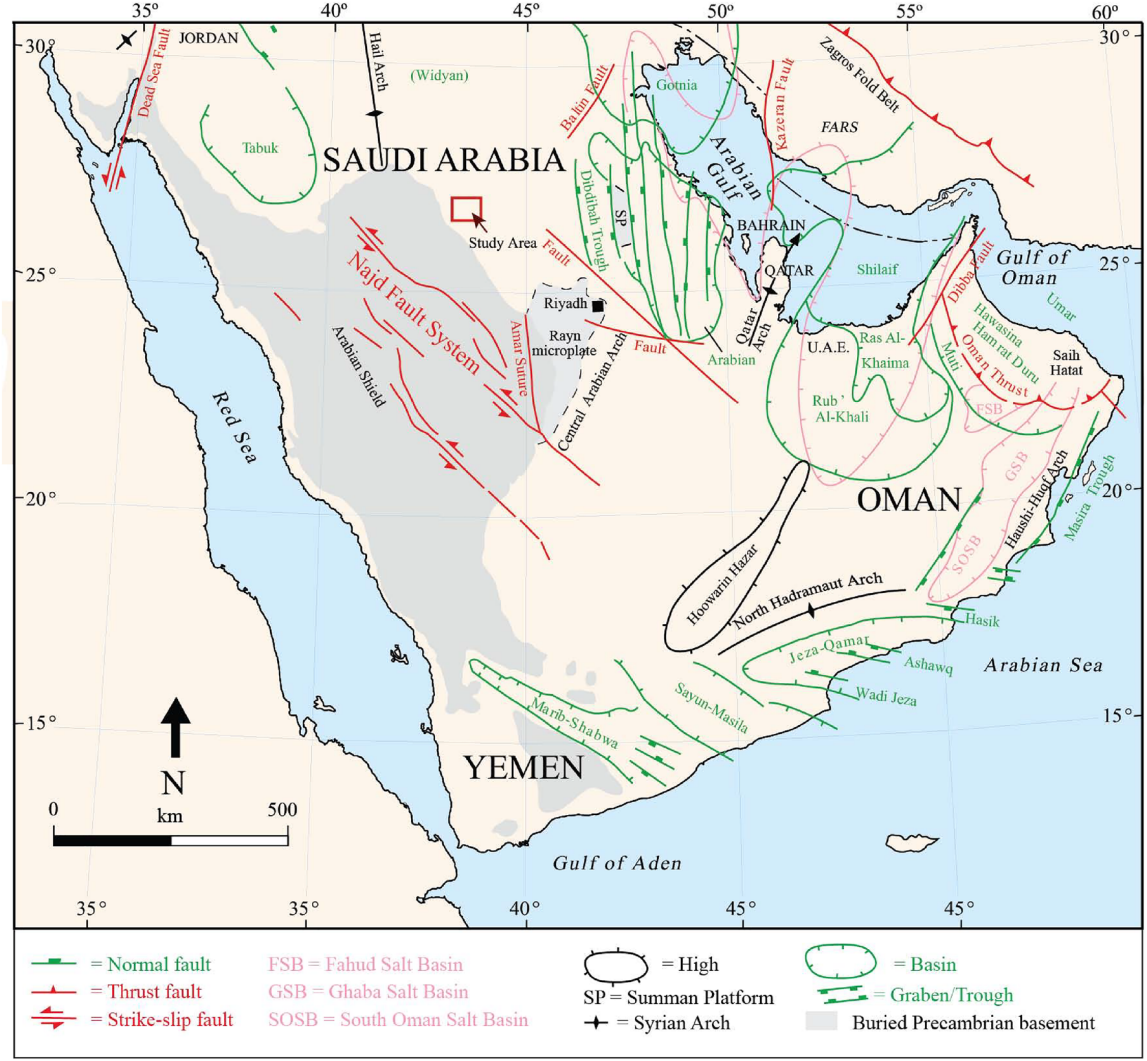
The location of the study area on a structural map of the Arabian Peninsula; modified after Ziegler^33^.

**Figure 2:**
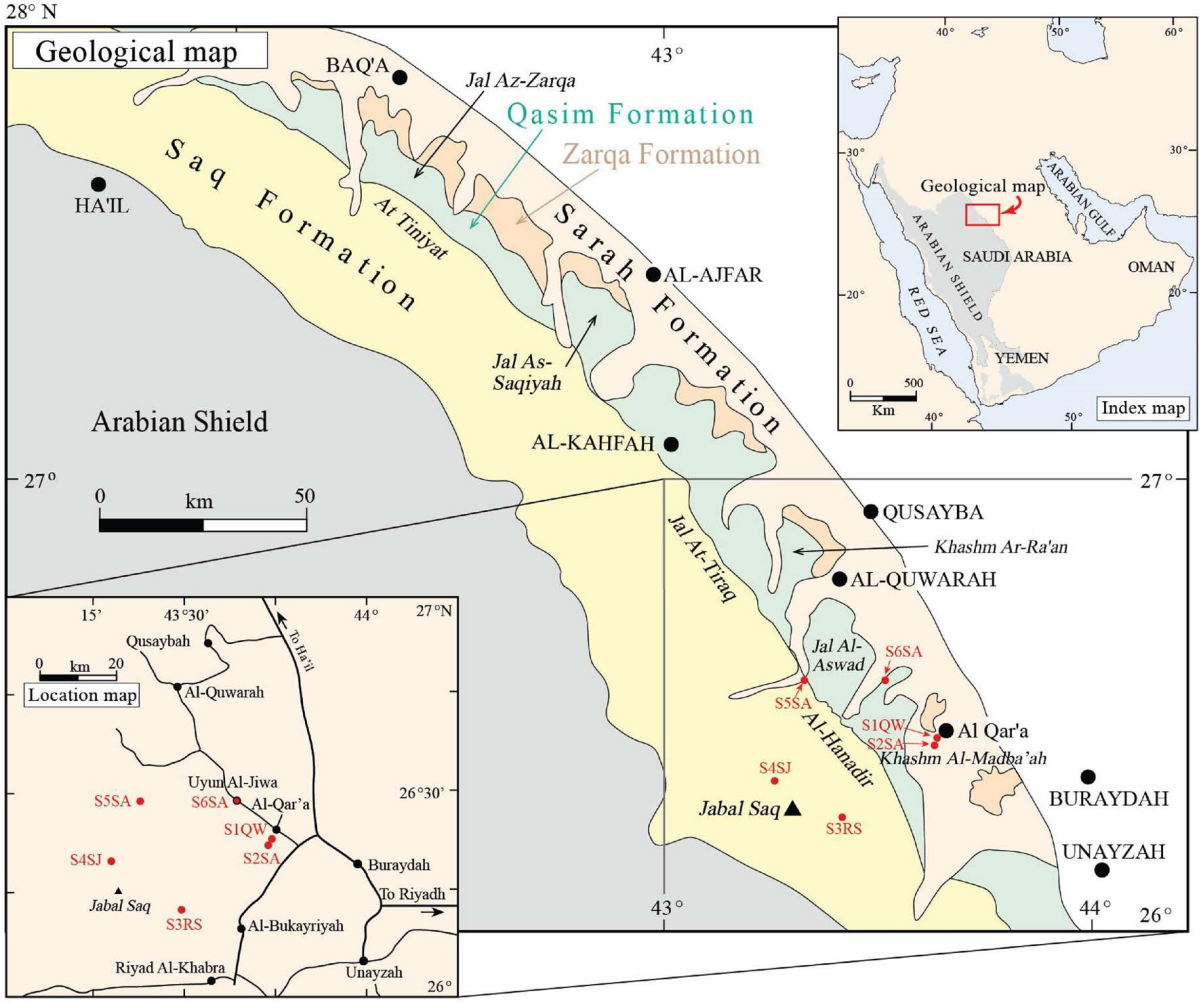
Index, location, and geological maps of the studied locations in the Qasim region; modified after Senalp and Al-Duaiji^31^.

**Figure 3:**
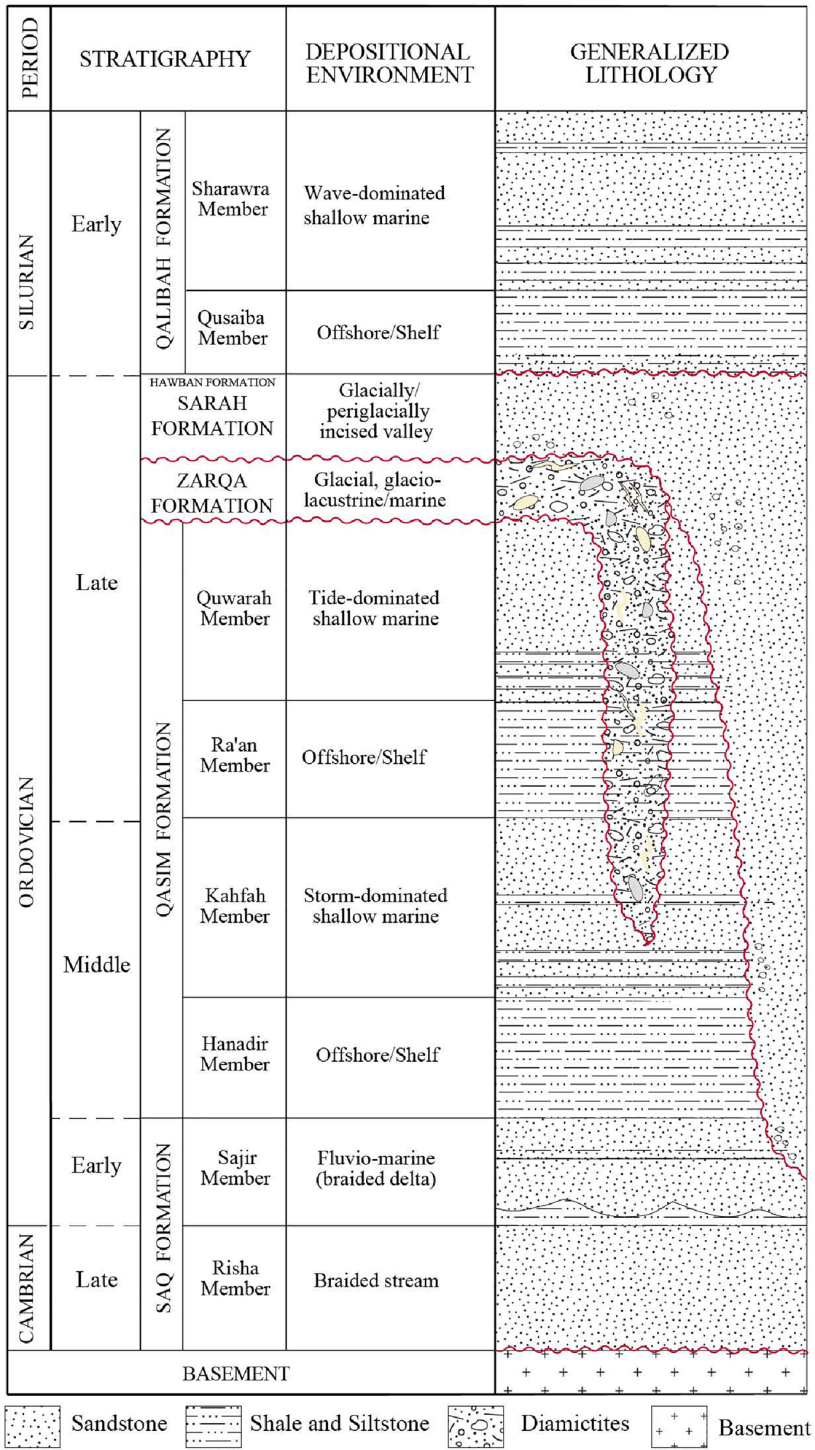
Stratigraphic column of the Cambrian and Ordovician siliciclastic sequences and their depositional settings; modified after Senalp and Al-Duaiji^31^ and Vaslet^34^.

The Cambrian–Ordovician sandstones in Al Qassim Province (west, west north, and west south of Buraydah), a central region of Saudi Arabia (Figs. 1, 2), include, from older to younger, Risha and Sajir Members of the Saq Formation, Kahfah and Quwarah Members of the Qasim Formation, and Zarqa, Sarah, and Hawban Formations^30,31^ (Fig. 3). The Risha Member unconformably overlies the Proterozoic basement rocks and is characterized by coarse-grained braided sandstones at the lower part and medium-grained deltaic sandstones at the upper part, which is also reddish, beige, brownish, whitish sandstones with cross-bedding and micaceous contents^30,32^. The Sajir Member, which conformably overlies the Risha Member, is mainly composed of tidal flat and coastal plain deposits, characterized by whitish, brownish, fine- to medium-grained sandstones, micaceous siltstones preserving *Cruziana* traces in the lower part, and siliceous and ferruginous cement in the uppermost part^32^.

The Early Paleozoic sandstones include the storm- and tidal-dominated sandstones of the Kahfah and Quwarah Members of the Qasim Formation, respectively^31^. The Kahfah Member is characterized by arenitic, fine- to medium-grained sandstone with clayey and silty layers at the lower part^32^. It is also characterized by *Skolithos*, fine-grained sandstone with silty and micaceous contents at the upper part^32^. The Quwarah Member is characterized by interbedded silty claystone and ferruginous and micaceous, fine- to medium-grained sandstones^33–35^. It also contains bioclasts^31^ and is commonly channelized by the Upper Ordovician Glaciogenic Deposits (UOGD)^35,36^.

The UOGD in Al Qassim Province, including Zarqa, Sarah, and Hawban Formations, are age-equivalent to Sanamah Formation in southwest Saudi Arabia. They are mainly sandstone, fine-grained, and diamictites^35–37^. In Al Qassim Province, the thickest sandstones are present in Sarah and Hawban Formations (90–300 m), ranging from fine- to coarse-grained, moderately, and poorly sorted^32,34,36^. Ferruginous cementation and surfaces have been reported from the sandstone facies of the UOGD^34,35,37^.

## Methodology

Six outcrop sites were investigated to describe the sedimentological facies, with the primary objective of characterizing the geometry and lateral and vertical distribution of ferruginous deposits (Figs. 1 and 2, Table 1). The locations are numbered sequentially and named after their respective formation names, as follows: Site 1 Quwarah Member (S1QW), Site 2 Sarah Formation (S2SA), Site 3 Rish Member (S3RS), Site 4 Sajir Member (S4SJ), Site 5 Sarah Formation (S5SA), and Site 6 Sarah Formation (S6SA). During the field investigation, a total of 70 representative samples were selected for laboratory analyses, which included several techniques such as thin-section petrography, micro-X-ray fluorescence (µXRF), X-ray powder diffraction (XRD), Quantitative Evaluation of Minerals by Scanning Electron Microscopy (QEMSCAN), and computerized tomography (CT). We conducted bulk mineralogical analysis of 57 samples using XRD, and surface mineralogy analysis of 52 samples using QEMSCAN (Supplementary data). By integrating the results of these analyses, we validate the data obtained from each instrument.

**Table 1:** Summary of information related to the studied sections.

Stop	ID	Age	Group	Formation	Member	Lat	Long	Remarks
1	S1QW	Caradocian	Tayma	Qasim	Quwarah	26°23′15.47″N	43°45′41.74″E	Khashm al Madba'ah
2	S2SA	Hirnantian	Tabuk	Sarah		26°22′59.35″N	43°45′12.85″E	Khashm al Madba'ah
3	S3RS	Late Cambrian	Tayma	Saq	Risha	26°13′2.66″N	43°27′7.96″E	Roadcut
4	S4SJ	Early Ordovician	Tayma	Saq	Sajir	26°18′20.08″N	43°17′4.87″E	Jabal at Tays
5	S5SA	Hirnantian	Tabuk	Sarah		26°34′17.11"N	43°22′1.78″E	Jal al Aswad
6	S6SA	Hirnantian	Tabuk	Sarah		26°33′42.93″N	43°35′45.44″E	Gaf Al Jewa

To evaluate the distribution of various elements, including Fe, Ca, Si, S, Al, K, Mg, and Mn, we selected a set of rocks to be slabbed. The geochemical distribution of these samples was analyzed using a non-destructive µXRF system. The slabbed samples were scanned directly without any polishing via the µXRF, and the system was operated using procedures already described in the literature^38,39^. This approach allowed us to accurately determine the distribution of these elements within the rocks, providing valuable insights into their geochemical properties.

For thin-section petrography, the selected samples were prepared with a blue epoxy to aid in identifying pores and pore types. We utilized a petrographic microscope to determine the detrital and authigenic components of the samples. Furthermore, we examined the grain texture of the samples, including their size and sorting. This approach allowed us to gain a detailed understanding of the mineralogical and textural properties of the rocks, which in turn provided valuable insights into their depositional and diagenetic history.

To assess the bulk and surface mineralogy of the samples, we employed XRD and QEMSCAN, respectively. The XRD technique was utilized to identify and quantify the bulk mineralogical phases in the studied samples. Following pulverizing the samples, the XRD instrument detected the mineral phases identified by mineral databases. The concentration of each mineral phase was then estimated in automated full pattern summation mode. The XRD approach allowed us to determine the bulk mineralogical composition of the samples, providing valuable insights into their geological history and depositional settings. The QEMSCAN technique, on the other hand, was employed to assess the surface mineralogy of the samples, producing detailed mineralogical maps of the samples and allowing us to gain a deeper understanding of their microstructural and textural properties.

The QEMSCAN technique is a sophisticated petrographic tool that combines a Scanning Electron Microscope (SEM), Energy-Dispersive Spectroscopy, and Species Identification Protocol (SIP) to produce quantitative mineralogical maps from rock samples^40–42^. We employed this technique to assess the surface mineralogical composition of a select set of thin sections and core plugs representing various ferruginous deposits. Before the analysis, the selected and prepared samples were carbon-coated using a Quorum EMS 150R ES. The standard QEMSCAN setup was utilized^40–42^, and the system was operated with an X-ray beam voltage of 15 kV and a beam current of 10 nA (± 0.05). The Field Image Scan mode was chosen, covering an area of 1 cm^2^ with a 5-μm point spacing. The data was processed using the iDiscover software, where a series of processors, including field stitching, granulator, and boundary phase, were applied to generate surface mineralogical maps.

In addition, we conducted CT scans on several chosen samples that contained ferruginous deposits to examine their three-dimensional morphology and compare them with similar deposits found in other parts of the world. The data had a resolution of 0.5 mm, and we utilized open-source software, 3D Slicer 4.13^43,44^, to segment and visualize the CT scan data.

## Results

### Sedimentological description

Six locations west of Buraydah town were visited to investigate the distribution of ferruginous deposits in the Early Paleozoic sandstones (Figs. 1 and 2), including the Late Cambrian to Early Ordovician Saq Formation, Risha (S4RS), and Sajir (S4SJ) Members located about 15 km southeast and 5 km northwest of Jabal Saq, respectively, the Caradocian Quwarah Member of the Late Ordovician Qasim Formation in Khashm al Madba'ah (S1QW), and the Late Ordovician (Hirnantian) Sarah Formation in Khashm al Madba'ah (S2SA), Jal al Aswad (S5SA), and Gaf Al Jewa (S6SA) (Table 1).

#### The Late Cambrian to Early Ordovician Saq Formation

**Figure 4:**
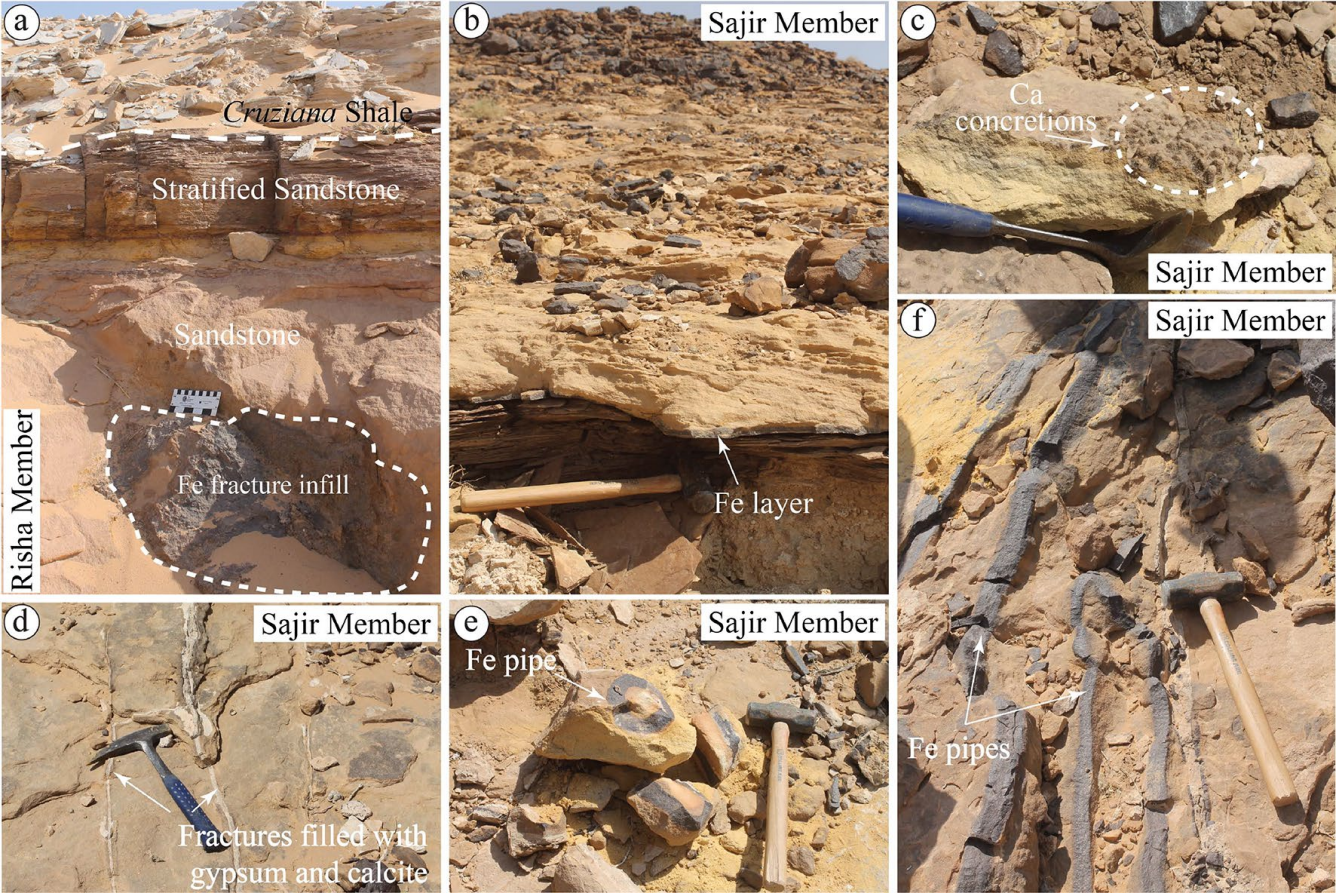
Sedimentary facies and ferruginous deposits observed in (**a**) Risha (S3RS), and (**b**–**d**) Sajir (S4SJ) Members of the Saq Formations. The former contains (**a**) ferruginous (Fe) fracture infill, while the latter contains ferruginous (**b**) layers, and (**e**,**f**) pipes, as well as (**c**) calcite (Ca) concretion, and (**d**) fracture infills. The ferruginous pipes were found at the top of the studied S4SJ section (**b**).

S3RS and S4SJ represent parts of the Risha and Sajir Members of the Saq Formation, respectively (Fig. 3). The studied sections contain relatively less abundant ferruginous deposits than the other sections of this study. S3RS and S4SJ sections are located around Jabal Saq (Fig. 2), with no mapped glaciogenic deposits^32^. The studied S3RS section, located about 15 km southeast of Jabal Saq, consists of beige, fine- to medium-grained, moderately sorted deltaic sandstone^32^ and represents the Risha Member's upper part overlain by *Cruziana* shale. The S3RS contains reddish and brownish stratified sandstone layers at the top (~ 20 cm) and a fracture infill of ferruginous deposits extended to the middle part of the section (Fig. 4a).

The S4SJ section, located around 5 km northwest of Jabal Saq, represents the basal part of the Sajir Member. It contains mainly shallow-water sandstones^32^. The S4SJ contains a higher abundance of ferruginous deposits than in S3RS. The encountered ferruginous deposits in S4SJ occur as undeformed layers separating silty sandstones from the overlain cross-bedded, fine- to medium-grained, occasionally coarse-grained sandstone at the lower part of the section (Fig. 4b). Calcite cement occurring as concretions (Fig. 4c) and filling fractures (Fig. 4d) are commonly found in the middle part of the section. At the topmost of the section, ~ 1 m long ferruginous pipes with a diameter ranging between 10 to 15 cm were observed (Fig. 4e,f).

#### The Late Ordovician Quwarah Member

**Figure 5:**
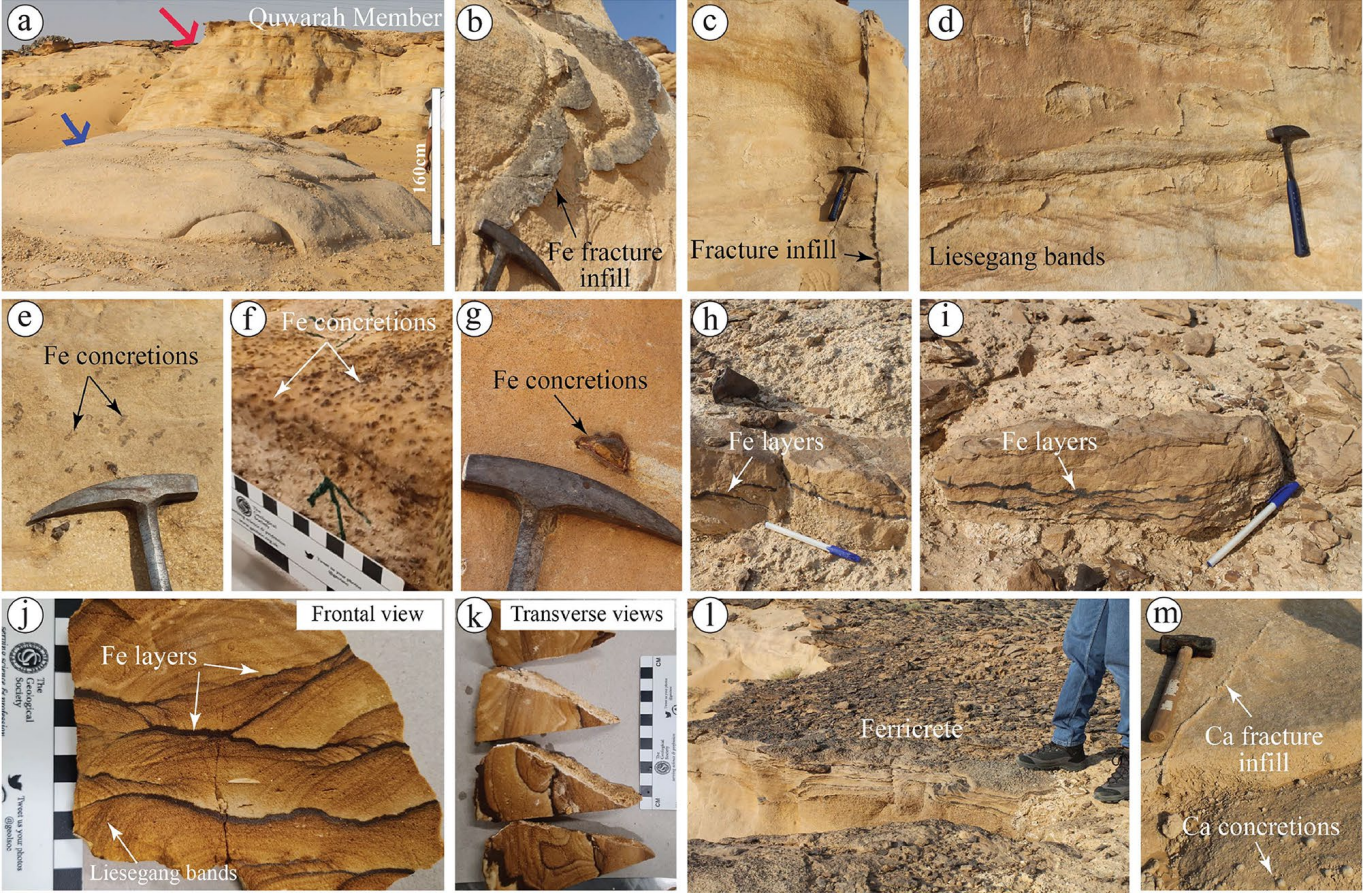
Sedimentary facies and ferruginous deposits were observed in (**a**) the Quwarah Member (S1QW) of the Qasim Formation. Note that the red arrow indicates sandstones with ferruginous deposits. In contrast, the blue arrow indicates sandstones with calcite concretions, and no ferruginous content is observed. (**b**,**c**) ferruginous (Fe) fracture infill, (**d**) cross-bedded sandstone associated with liesegang bandings. (**e**–**g**) isolated ferruginous concretions with various sizes. (**h**–**k**) deformed ferruginous layers and liesegang bandings. Note the deformed ferruginous layers show various distribution patterns as observed in (**j**) a frontal view and (**k**) transverse views. (**l**) deformed ferruginous layers forming ferricretes. (**m**) calcite concretions associated with calcite (Ca) fracture infill in a bleached sandstone.

The Quwarah Member of the Qasim Formation in Khashm al Madba'ah (S1QW) is mainly beige to whitish ("bleached"), fine- to medium-grained, well-sorted tidal marine sandstones^35,45^. It represents the basal part of the Quwarah Member, whose type section is located northwest of Al Quwarah town (26° 49′ 40.0″ N, 43° 24′ 38.0″ E)^32^. In S1QW (Table 1), the beige to light-beige sandstone contains dark brownish ferruginous deposits, while the whitish to off-whitish part includes nodular calcite (Fig. 5a).

The ferruginous deposits are distributed in various forms: (1) fracture infills (Fig. 5b,c), (2) light brownish arcuate bands (Fig. 5d) in the lower and middle parts of the studied section, (3) isolated, spaced, millimeter-scaled, irregular spheres (Fig. 5e–g), (4) deformed iron oxides layers with small arcuate bands (Fig. 5h–k, Supplementary Data), and (5) ferruginous duricrust at the top of the section. The arcuate bands are akin to those described as liesegang bands in several studies^46–48^, while the isolated spheres and the Fe-rich duricrust resemble the features of the ferruginous concretions and the ferricrete, respectively. Across S1QW, the liesegang bands (Fig. 5d,j,k) and the deformed iron oxide layers (Fig. 5h–k) occur in various sizes and orientations. In S1QW, calcite nodules or concretions are present in the whitish sandstones that are only a few meters away from the sandstones with ferruginous deposits (Fig. 5a,m). The calcite concretions range from about 5 mm to about 3 cm and are associated with calcite fractures in the whitish sandstones.

#### The Late Ordovician "Hirnantian" Sarah formation

##### Khashm al Madba'ah (S2SA)

**Figure 6:**
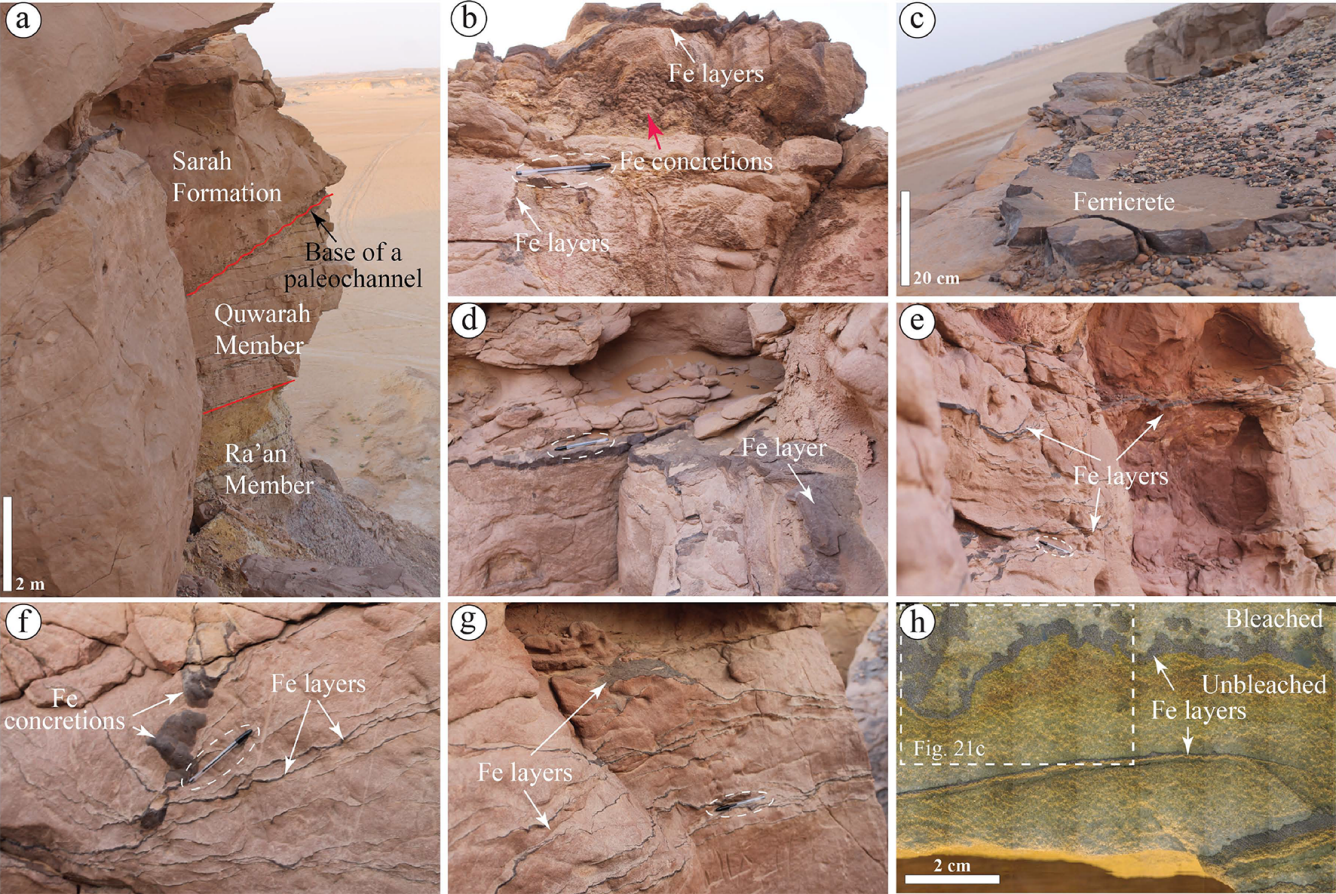
Sedimentary facies and ferruginous deposits observed in (**a**) the Sarah Formation at S2SA, which deeply cut the older Quwarah and Ra'an Members of the Qasim Formation. (**b**–**g**) Ferruginous (Fe) layers, concretions, and ferricretes were observed in S2SA. (**f**) Ferruginous layers and amalgamated concretions meet and the former is extended and distributed laterally (**g**). (**h**) A slabbed rock sample shows the distribution of ferruginous layers, bleached and unbleached areas in S2SA.

In the Khashm al Madba'ah, around 1 km southwest of S1QW (Figs. 1 and 2), the Hirnantian Sarah formation (S2SA) unconformably overlies the Quwarah Member of Qasim formation (Fig. 6a). It is mainly composed of beige, fine- to medium-grained, occasionally coarse-grained sandstones with ripple marks and ferruginous surfaces deposited in a glaciofluvial setting^32,34,35,37^.

Such sandstones contain various ferruginous deposits in their middle and upper parts. In the upper part, ferruginous concretions (Fig. 6b) and ferricretes were observed (Fig. 6c). Towards the middle part, deformed ferruginous layers (forming as surfaces) (Fig. 6d–h) occasionally associated with ferruginous concretions were also observed (Fig. 6f). A slabbed rock sample from S2SA with ferruginous layers shows sandstones with various colors along with regular to irregular shapes, indicating bleach and unbleached sandstones (Fig. 6h).

##### Jal al Aswad (S5SA)

**Figure 7:**
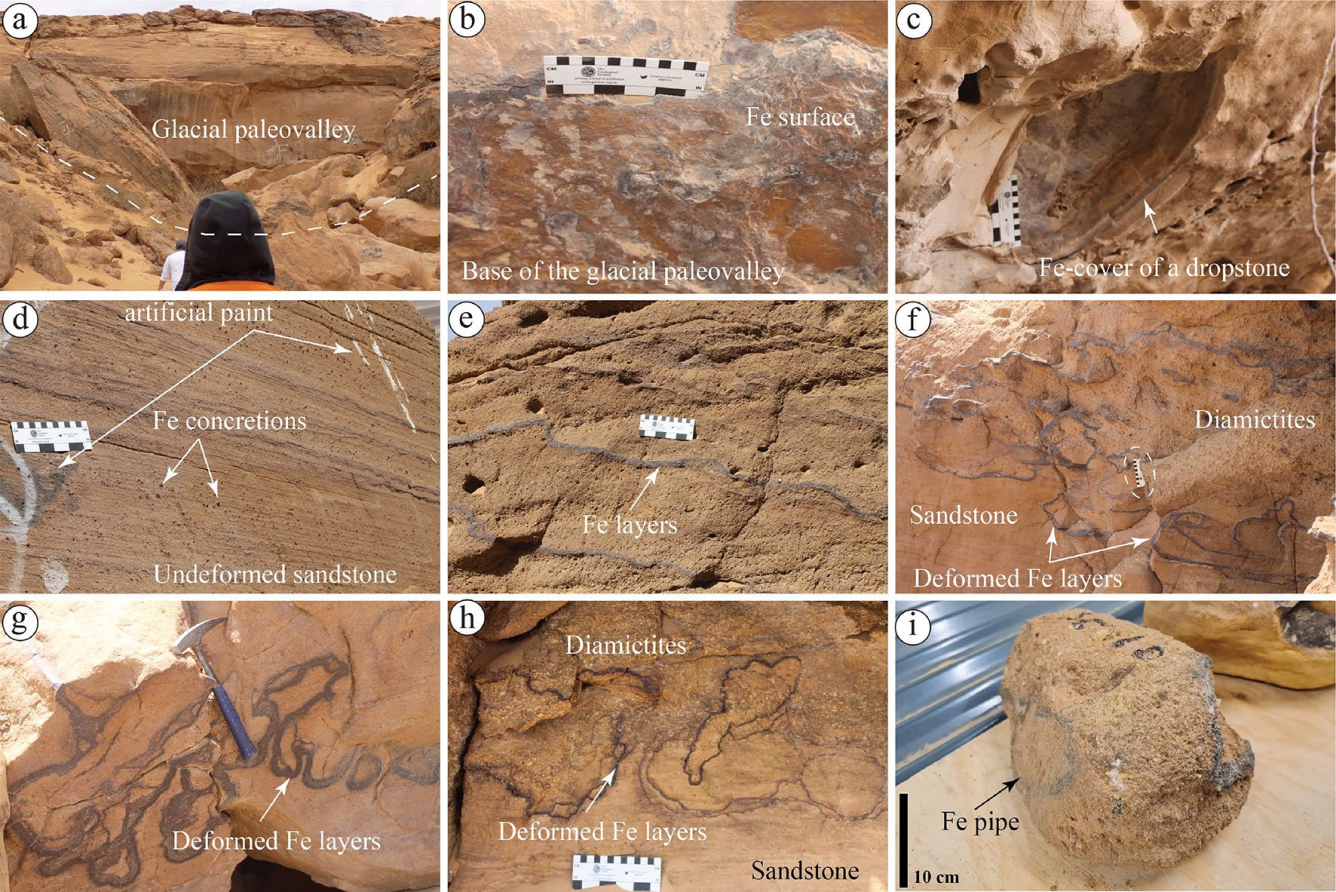
Sedimentary facies and ferruginous deposits observed in (**a**) the Sarah paleovalley at S5SA. (**b**) Ferruginous surfaces at the base of the paleovalley are present in a localized area and cover (**c**) a large spherical shape, possibly dropstone. (**d**) Undeformed and parallel layers of sandstone with millimeter-scale ferruginous (Fe) concretions. (**e**–**h**) Defamed ferruginous layers in very coarse-grained sandstones and diamictites. (**i**) A ferruginous pipe was found in the diamictites facies of S5SA.

The Sarah Formation in its type locality, Sarah Ridge (S5SA), has a more heterogeneous grain texture than S2SA, which is 20 km southeastward (Figs. 1 and 2). It mostly contains clast-supported diamictites and medium- to coarse-grained sandstones of subglacial and glaciofluvial settings, filling a paleovalley—named Sarah paleovalley^32,34^ (Fig. 7a). The Sarah paleovalley in S5SA is found cutting the older Qassim Formation, resulting in forming an unconformable contact between the Sarah Formation and the underlying Handir Member of the Qassim Formation. The abundance of ferruginous deposits in S5SA is relatively high compared to the other studied formations. At the basal part of the paleovalley, ferruginous surfaces/layers are observed (Fig. 7b). It was also covering a large spherical material expected to be a "dropstone" (Fig. 7c). In addition to the recorded ferruginous surfaces in S5SA, small concertations, and thin ferruginous layers were found along the bedding planes in undeformed sandstones (Fig. 7d). They are also present in deformed sandstones and clast-supported diamictites, forming ferruginous surfaces with various shapes (Fig. 7e–h). The glacial diamictites were found to be having relatively higher ferruginous content than the other encountered sandstones in S5SA. In the same context, the sediments on the paleovalley's sides had more ferruginous content than those found along the axis. Pipe concretions, similar to the ones observed in S4SJ in terms of dimensions but different in grain texture, were also observed in S5SA (Fig. 7i).

##### Gaf Al Jewa (S6SA)

**Figure 8:**
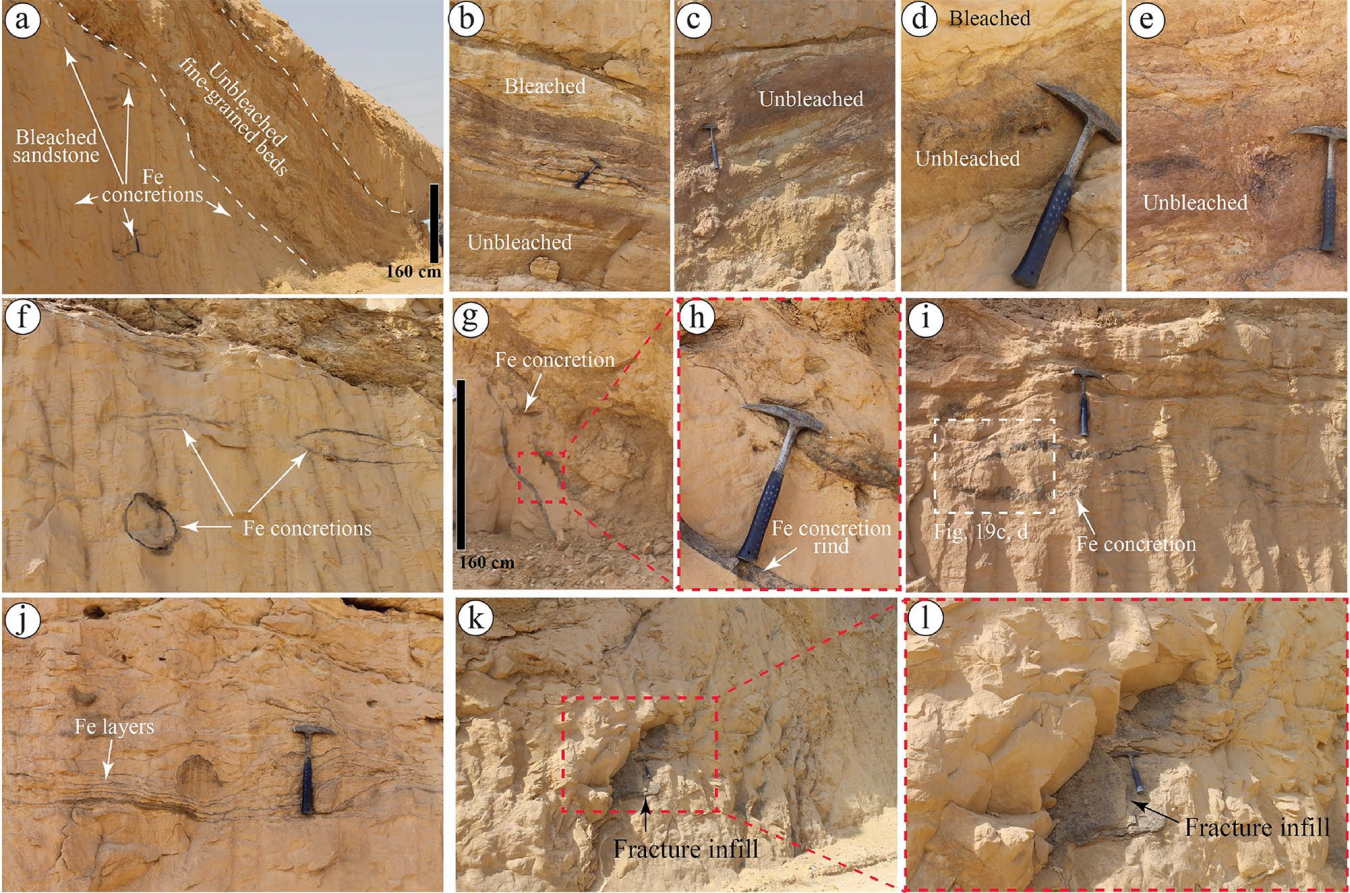
Sedimentary facies and ferruginous deposits observed in the Sarah Formation at S6SA. (**a**) Outcrop of unbleached fine-grained beds (mainly siltstone), and bleached sandstones, where ferruginous (Fe) rinded concretions are distributed. (**b**–**e**) various colors of unbleached siltstones. (**f**–**i**) ferruginous rinded concretions. (**j**) ferruginous layers. (**k**,**l**) fractures infill of mixed ferruginous, calcareous, and gypsiferous deposits.

At the location referred to as Gaf Al Jewa (S6SA), also known as Jal al 'Aqabah on the geological map (Fig. 2) and Rawd Al-Jawa or Rawdal Uyun paleovalley in journal publications^23,34^, the Sarah Formation is distinguished by siltstone and fine-grained sandstone beds of glaciofluvial origin. These fine-grained beds are mainly unbleached, while the sandstones are mainly bleached (Fig. 8a). The unbleached part is deformed and occurs with different colors, including greenish brown, light brown, dark brown, and black (Fig. 8a–e). The bleached part is mainly medium-grained to coarse-grained sandstone with various ferruginous deposits, including concretions, layers, and fracture infill. The ferruginous concretions occur with different shapes and dimensions ranging from spherical to ellipsoidal, and rind thicknesses reach up to 4 cm (Fig. 8f–i). These concentrations are disconnected and filled with the same host sandstones (Fig. 8h). Multiple thin ferruginous layers with deformed patterns (Fig. 8j) and fractures filled with goethite, gypsum, and calcite minerals (Fig. 8k,l) are also observed in the in S6SA.

### Geochemical composition

**Figure 9:**
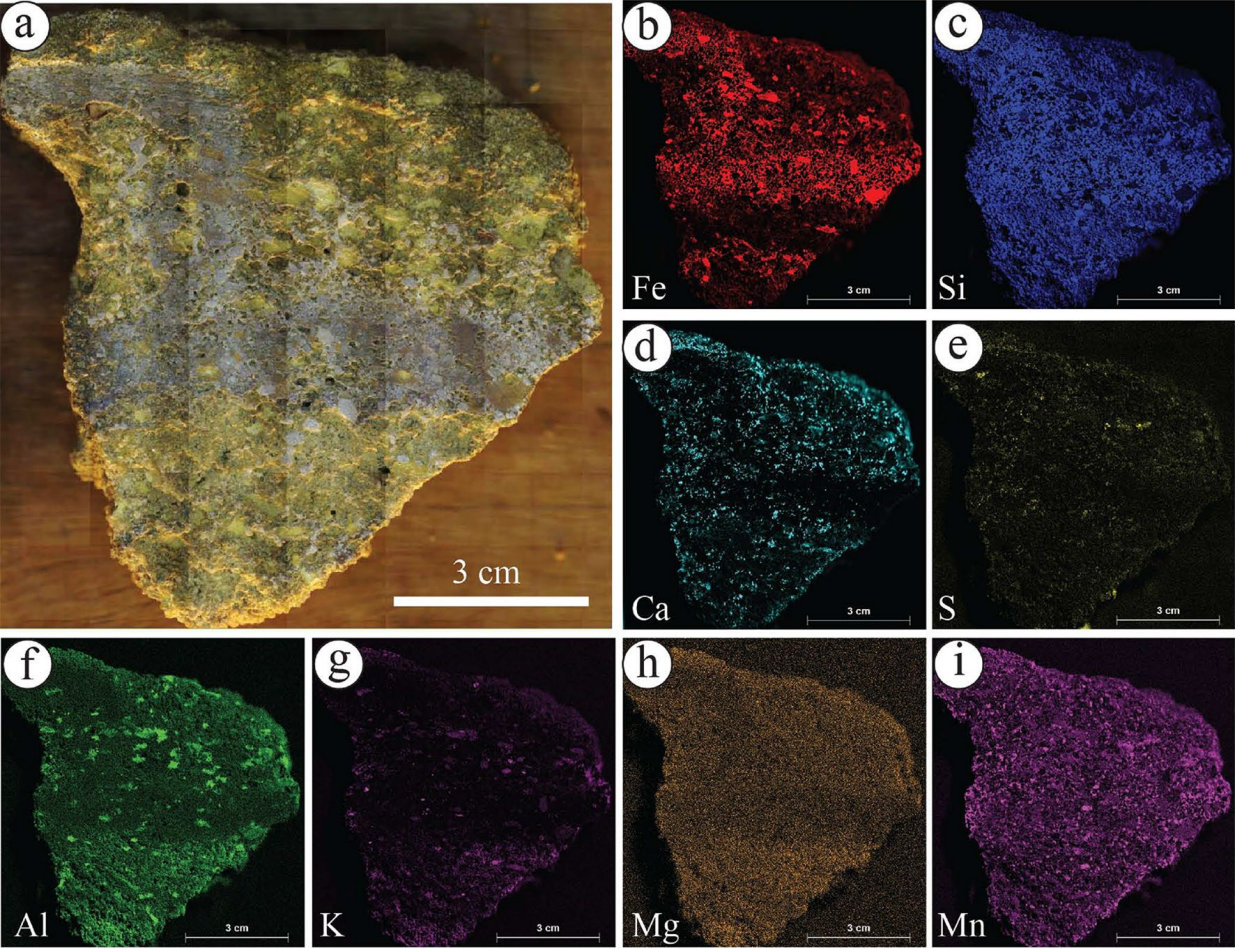
(**a**) Ferruginous rinded concretion in the diamictites facies of S5SA and (**b**–**i**) its geochemical maps. Note that Fe distribution is associated with increases in Ca, Al, and Mn elements.

**Figure 10:**
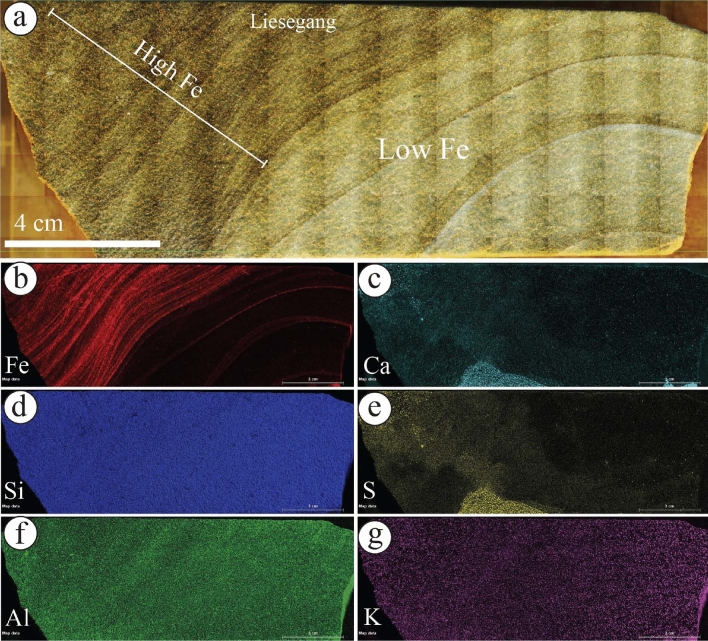
(**a**) Liesegang bandings of S1QW and (**b**–**g**) their geochemical maps. Note that Fe distribution is only present in the darker areas of the bandings, while Ca and S elements occur in localized spots in the lighter areas indicating the occurrence of gypsum minerals.

**Figure 11:**
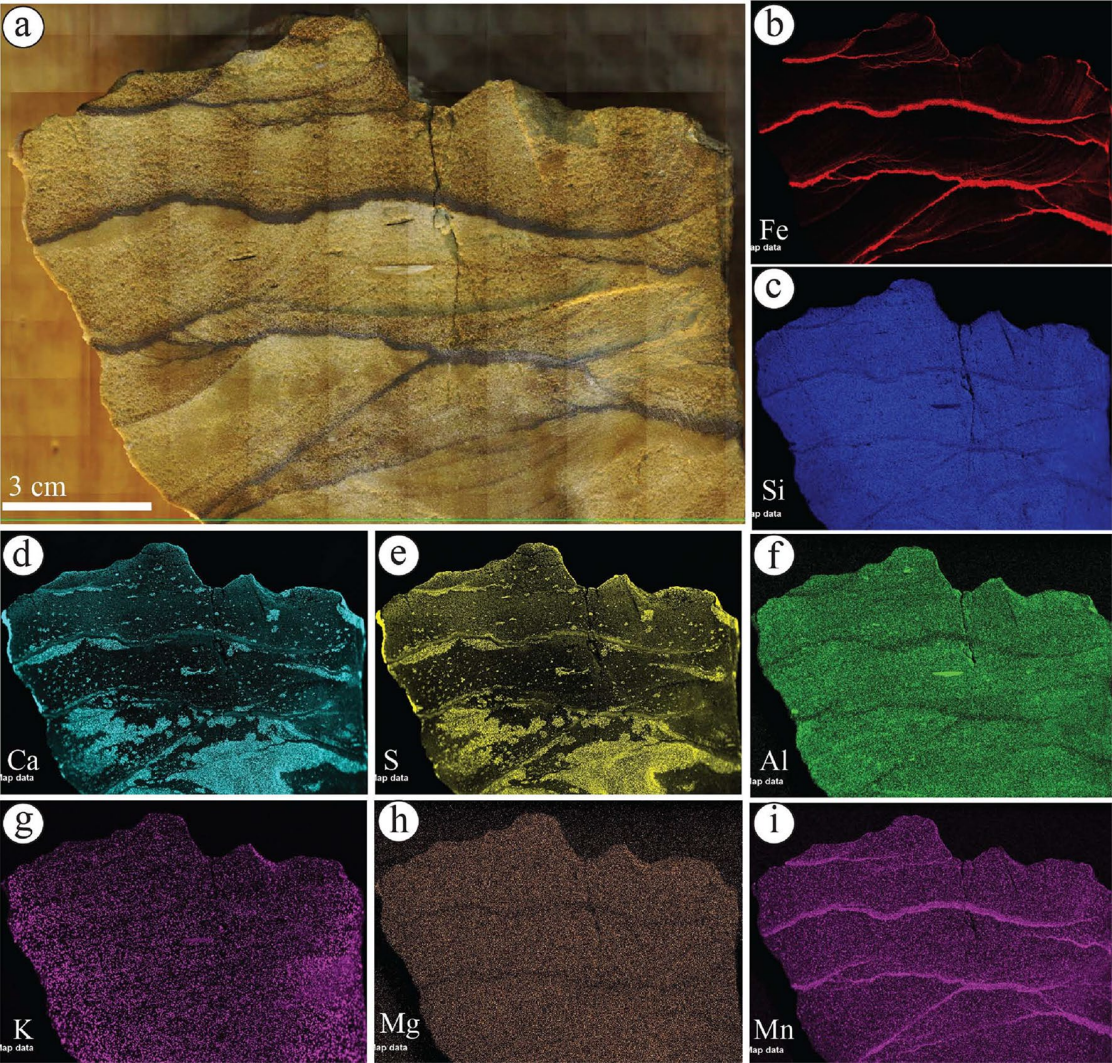
(**a**) Ferruginous layers of S1QW and their geochemical maps of. Note that Fe and Mn contents are mainly concentrated in the deformed black layers. The Ca and S elements are indicative of gypsum minerals.

The geochemical mapping of selected samples showed that the Fe concentration is increased in the rinds of ferruginous concretions observed in the diamictites of the Sarah Formation (S5SA) (Fig. 9a–c). It is occasionally associated with Ca concentration (Fig. 9d) and other elements with various concentrations (Fig. 9e–i). In the liesegang bandings facies of S1QW (Fig. 10a–g), the Fe concentration decreases towards the bleached part (Fig. 10a–b). It shows that the darker area represents the highest concentration of Fe, which increases with the increasing number of liesegang bandings. Ca and S concentrations are present but mainly in the bleached area away from the liesegang bandings (Fig. 10c, e). The deformed layers observed in S1QW (Fig. 11a–i) are characterized by Fe concentration (Fig. 11b) associated with Mn concentration (Fig. 11i). Ca and S concentrations are present in the host-bleached sandstones in addition to Al (Fig. 11f) and K elements (Fig. 11g).

**Figure 12:**
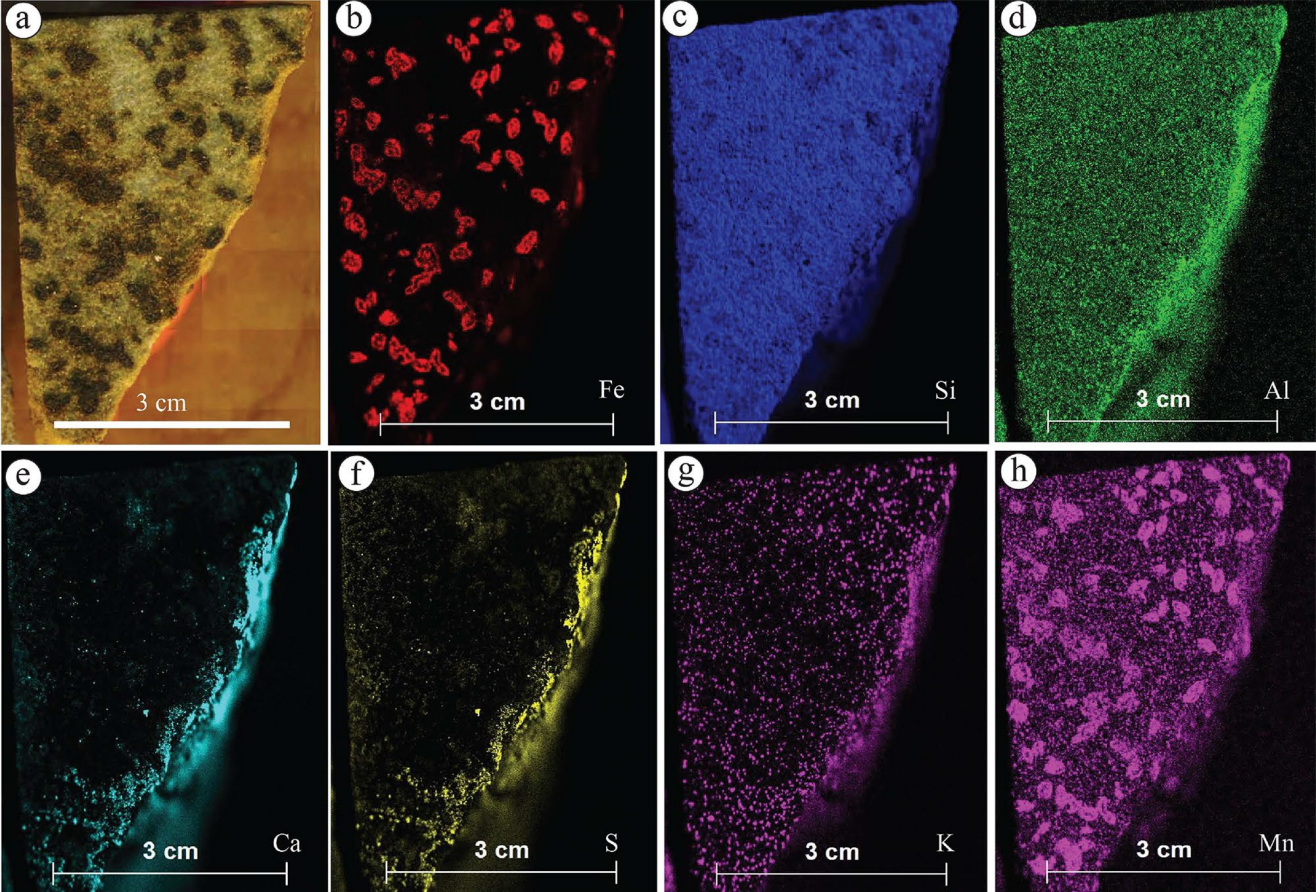
(**a**) Isolated ferruginous concretions observed in S1QW and (**b**–**h**) their geochemical maps. Fe and Mn elements represent the main components of these concretions. Ca, S, and Al elements increased due to artificial materials placed under the sample.

**Figure 13:**
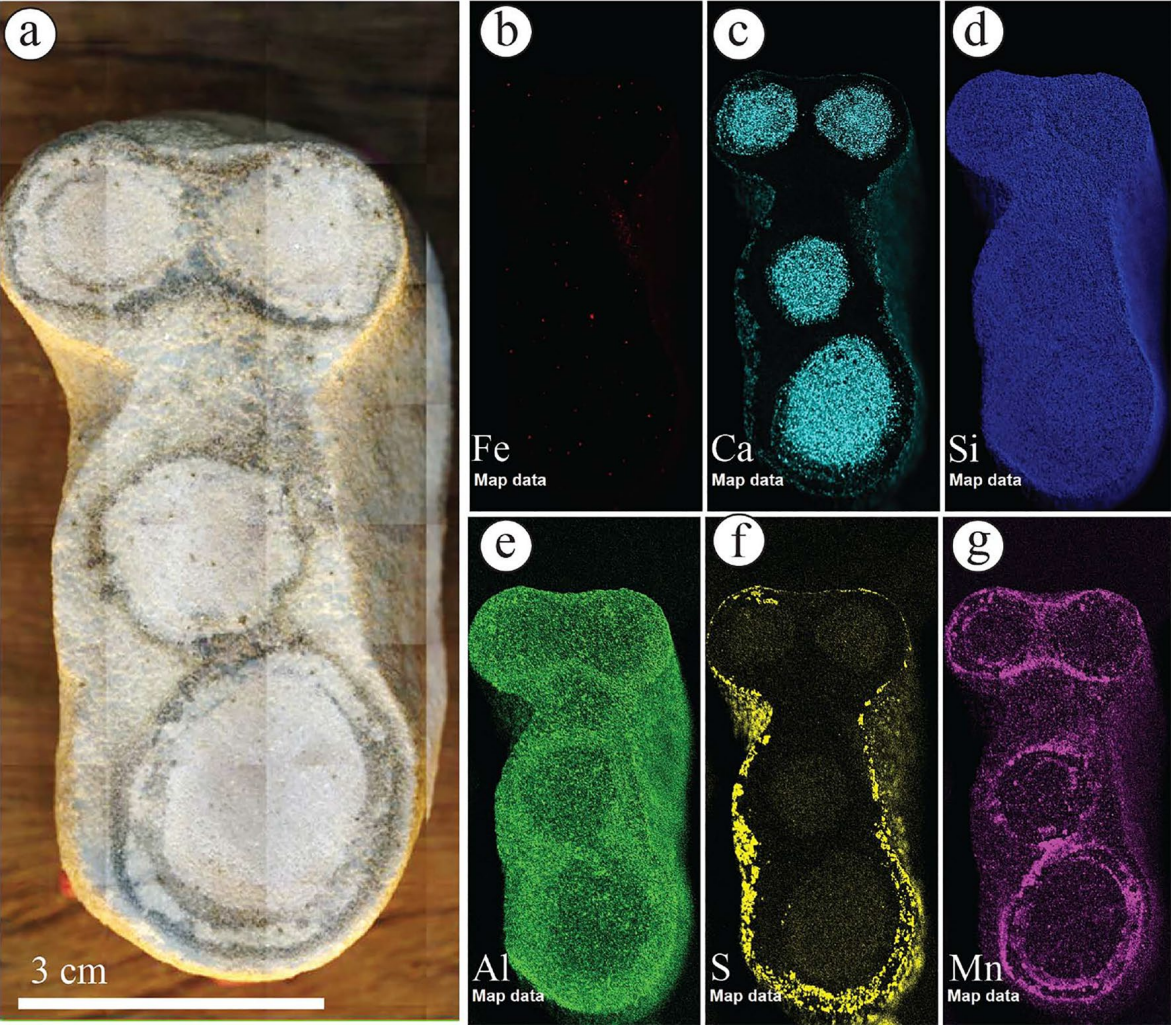
(**a**) Calcite concretions observed in S1QW and (**b**–**g**) their geochemical maps. Fe is absent, while Mn present as a double-coating material for calcite-rich sandstone, representing the calcite concretions. Al occurs in the host sandstone, but it decreases as Ca increases. S increased due to an artificial material placed under the sample.

The ferruginous concretions (Fig. 12a–h) are mainly composed of Fe (Fig. 12b) and Mn (Fig. 12h) elements, generally occurring without Ca and S concentrations (Fig. 12e,f). In contrast, the calcite concretions (Fig. 13a–g) show no presence of Fe (Fig. 13a,b), while Ca and Mn are commonly found at the center and the surroundings of the calcite concretions (Fig. 13c and g, respectively). The Al element is concentrated more in the host rock and decreases toward the center of calcite concretions (Fig. 13e).

### Mineralogical composition

**Figure 14:**
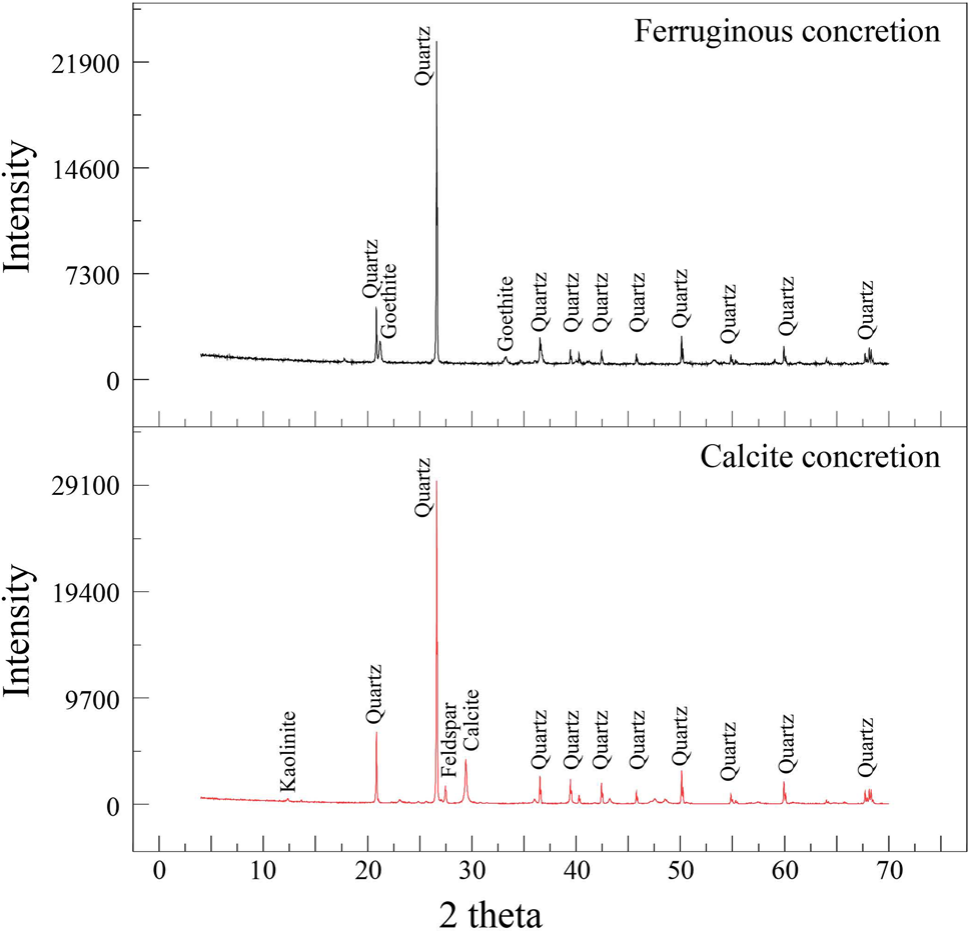
XRD peaks of ferruginous and calcite concretions in the sandstone of S1QW.

XRD bulk mineralogical analysis of selected sandstone samples from the studied locations suggested that S1QW samples contain a relatively higher feldspar content than the other locations. The other studied sections are dominated by quartz. Goethite is the main mineral for ferruginous content (Fig. 14, Supplementary data), with an occasional presence of hematite in S3RS only (Supplementary data). The XRD analysis of the studied concretions shows that the ferruginous concretion does not contain any calcite, and the calcite concretion does not contain any iron oxide minerals (Fig. 14). The clay mineral type in all sections is kaolinite, with an occasional association of illite.

**Figure 15:**
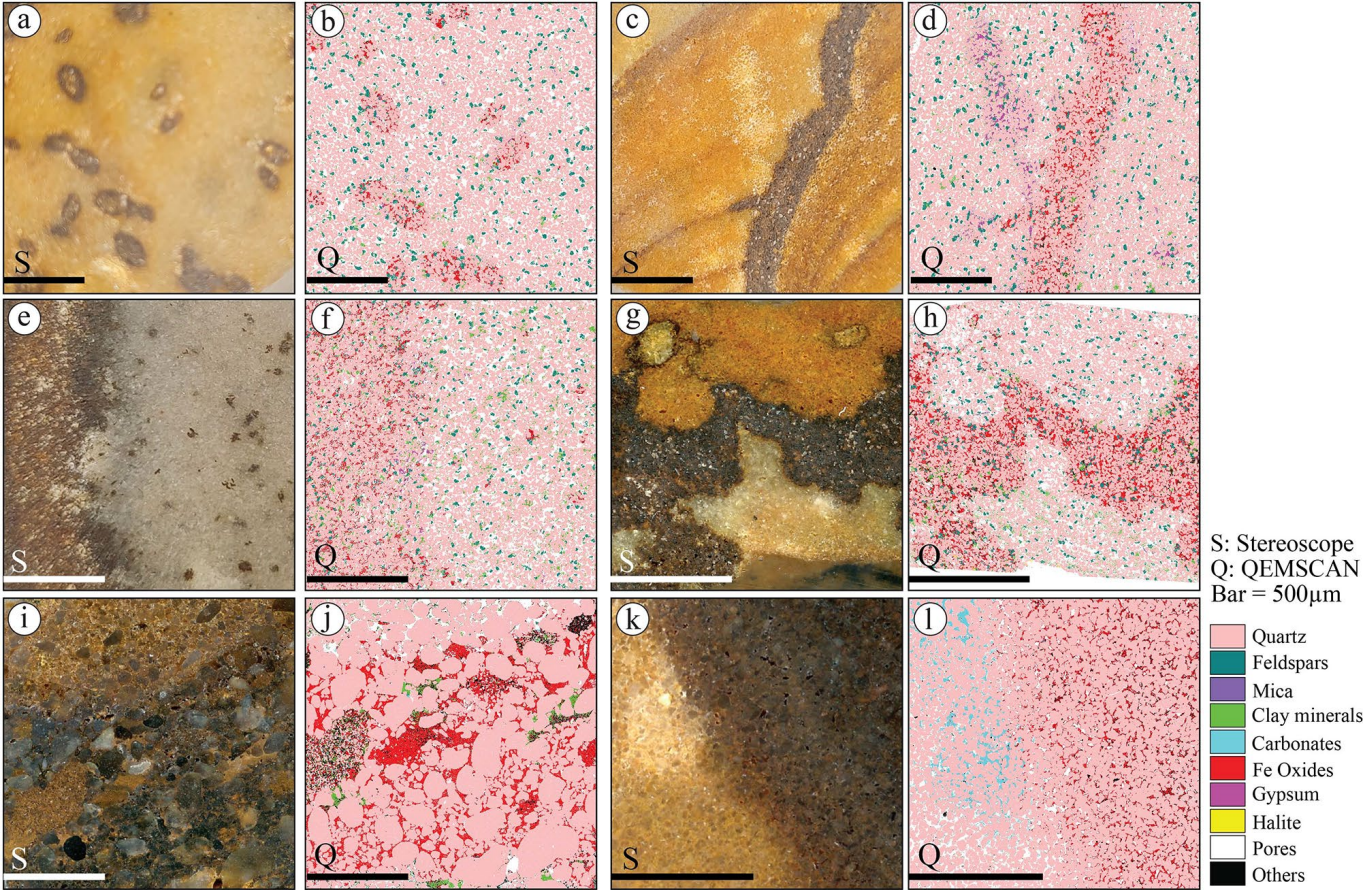
Stereomicroscope and QEMSCAN images of selected samples from ferruginous sandstone, including (**a**,**b**) ferruginous concretions, (**c**,**d**) ferruginous deformed layers in S1QW, (**e**,**f**) ferruginous fractures infill, (**g**,**h**) deformed ferruginous layers in S2SA, (**i**,**j**) ferruginous cement fill pore spaces in very coarse-grained sandstones and (**k**,**l**) ferruginous cement of S4SJ pipes associated with a calcite cement.

**Figure 16:**
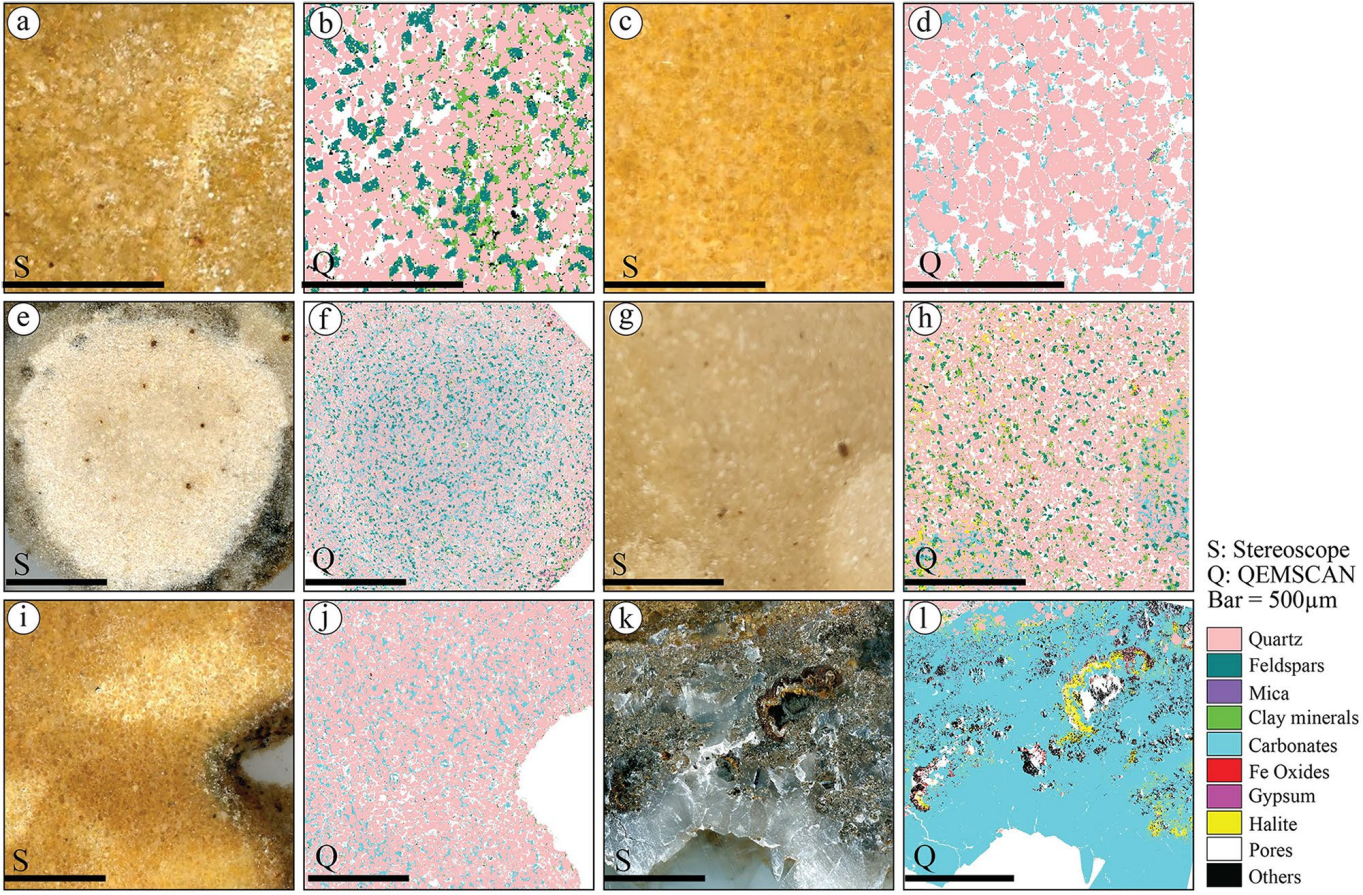
Stereomicroscope and QEMSCAN images of selected samples from studied sandstones and calcite cement, including (**a**,**b**) feldspar-rich porous sandstones of S1QW, (**c**,**d**) calcite cement in quartz-rich sandstone of S2SA, (**e**,**f**) calcite concretions, (**g**,**h**) calcite concretion in feldspar-rich sandstones, (**i**,**j**) calcite concretion of S4SJ, and (**k**,**l**) calcite crystals found as fracture infills of S6SA.

Surface mineralogical mappings of QEMSCAN show that ferruginous content in sandstone occurs as pore-filling cement distributed in various patterns (Figs. 15 and 16). The ferruginous concretions (Fig. 15a,b) occur as isolated pore-filling cement between quartz and feldspars associated with clay minerals. The ferruginous pore-filling cement between quartz and feldspars grains is associated with evaporite cement and clay minerals in the ferruginous layers of S1QW (Fig. 15c,d). However, the liesegang bandings associated with these layers (Fig. 15c) do not show any mineralogical variations in QEMSCAN images (Fig. 15d). Ferruginous fracture infills occur with a similar pattern of filling cement (Fig. 15e,f) in relatively higher porous sandstone. The QEMSCAN images of deformed ferruginous layers in S2SA (Fig. 15g,h) show the ferruginous cement follows the same pattern of deformation and no change in mineralogical composition between the two non-ferruginous areas (host rock and moat), which show different colors in the stereoscope images (Fig. 15g). The ferruginous pore filling cement in coarse-grained sandstone or diamictites is also present (Fig. 15i,j) and is associated with clay minerals. Non-ferruginous spots of such rocks are characterized by higher porosity than ferruginous-cemented spots (Fig. 15j). The QEMSCAN images of the ferruginous pipes in S4SJ show ferruginous pore-filling cement are associated with calcite cement in quartz-rich sandstones (Fig. 15k,l).

Generally, the S4SJ is the only studied section that shows a higher amount of calcite cement in feldspar- (Fig. 16a,b) and quartz-rich sandstones (Fig. 16c,d). It shows that the amount of calcite cement is higher in the quartz-rich part (Fig. 16d) than in the feldspar-rich sandstone, characterized by a relatively higher clay content (Figs. 5m, 16b). The calcite cement forming calcite concretions of different sizes reach 3 cm in diameter in S1QW (Fig. 16e) in both feldspar- and quartz-rich sandstones. Non-calcite cement spots of these sandstones contain clay minerals (Fig. 16h). However, the calcite cement in S4SJ occurs mainly in quartz-rich sandstone (Fig. 16i,j). In S6SA, clear calcite crystals (Fig. 16k,l) were found in the fracture infills and are associated with halite.

## Discussion

### Characteristics and distribution patterns of ferruginous deposits

The ferruginous deposits of the studied sections include concretions, layers, pipes, liesegang bandings, fracture infills, unbleached rocks, and ferricrete. From the field observations of this study and by following the classification schemes of the Navajo sandstone concretion by Potter et al.^9^, solid concretions with various sizes were observed in the upper parts of the shallow marine Quwarah Member (S1QW) of the Qasim Formation (Fig. 5e–g). On the other hand, rinded concretions with spherical and ellipsoidal shapes were found in the bleached sandstone of the Upper Ordovician glaciogenic Sarah Formation in Gaf Al Jewa (S6SA, Fig. 8f–i). The other studied sections of the Sarah Formation only show a few localized solid concretions with different sizes, i.e., from S2SA (Fig. 6b).

Ferruginous deformed and undeformed layers were observed in the Sarah Formation (S2SA, S5SA, and S6SA) and the upper part of the basal Quwarah Member (Fig. 5h–k, Supplementary Data). The deformation of such layers is expected to be related to the glacial processes. The ferruginous pipes were only found in S5SA (Fig. 7i) and the upper part of S4SJ (Fig. 4e,f). The liesegang bandings were only seen in S1QW (Figs. 5d,j,k, 10a, 11a). Ferruginous fracture infills were observed in S1QW (Fig. 5a,b) and S6SA (Fig. 8k,l). Babiker et al.^23^ investigated the types and mechanisms of fractures associated with the glaciofluvial deposits in S6SA, concluding that the ferruginous filling materials usually fill southeast-northwest trending fracture sets.

Unbleached siltstone and sandstones were observed only in S6SA and S1QW, respectively. Babiker et al.^23^ described the same siltstone beds in S6SA as slumped mudstone and siltstone and related the deformation separated by unconformities to the glacial movement of various glacial episodes. Ferricretes were observed in S5SA (Fig. 6c) and S1QW (Fig. 5l). The term ferricrete describes sedimentary deposits cemented by iron oxides and includes various definitions and classes^49–51^. Herein, the ferricrete definition of Furniss and Hinman^51^ is used. The authors defined the ferricrete as stratified sedimentary deposits cemented by iron oxides. The main differences between ferruginous layers/surfaces and ferricrete in this study are that the layers are thin surfaces (> 1 cm), usually multiple, and show localized distribution. In contrast, the ferricrete are thick individual layers showing hardcover or exposed surfaces. Generally, the distribution of the studied ferruginous deposits that show different patterns and characteristics in each location reflect various changes in depositional and post-depositional (diagenesis) processes that led to the final formation of such deposits.

The mineralogical composition of all the studied ferruginous deposits indicated that goethite is the most dominant mineral of the iron oxide minerals. Babalola et al.^52^ reported almost the same in the Wajid sandstone but also reported hematite in all their samples. The Wajid Sandstone is a Paleozoic siliciclastic succession located in southwestern Saudi Arabia^53,54^. It is composed of quartz-rich sandstones that likely originated from the basement of the Arabian Shield^55^. Aal and Nabawy^17^ did not report goethite and only reported hematite in the Wajid sandstone, with a relatively higher percentage than Babalola et al.^52^. Generally, iron oxide minerals occurred in the Paleozoic sequences as pore-filling cement with various sandstone types and are associated with clay minerals and occasionally with calcite cement.

### The source of Fe in the early Paleozoic sandstones

Many researchers have studied and reviewed iron oxides^18,52,56–58^ as they are widely spread on the Earth. Fe concentrations in ferruginous deposits range from 10 to 30% and have a geochemical affinity with Mn^58^. Rudmin et al.^16,59^ discussed the origins of Fe in both continental and marine environments. In continental settings, Fe is sourced through intensive weathering processes of igneous and metamorphic rocks^52,57,59^, transported via water in the form of colloids, which are tiny particles that remain suspended in the fluid, and ultimately deposited in sediments.

For the source of Fe in the Paleozoic Wajid sandstones, southwestern part of Saudi Arabia, Babalola et al.^52^ suggested several potential sources, including intense chemical weathering of mafic and intermediate rocks of the Arabian Shield, and hydrothermal activities related to the Najd fault system. In our study area, the abovementioned potential Fe sources that Babalola et al.^52^ suggested are reasonable. However, this study shows that most of the ferruginous deposits were observed in the Upper Ordovician Sarah Formation (S2SA, S5SA, and S6SA), mainly containing peri- and pro-glacial deposits. Several studies suggested that the peri-glacial deposits contain fragments of igneous rocks transported by glacial processes^60^. Other studies reported that the glacial deposits of the Sarah Formation are in unconfirmable contact with the basement rocks of the Arabian Shield^61^.

**Figure 17:**
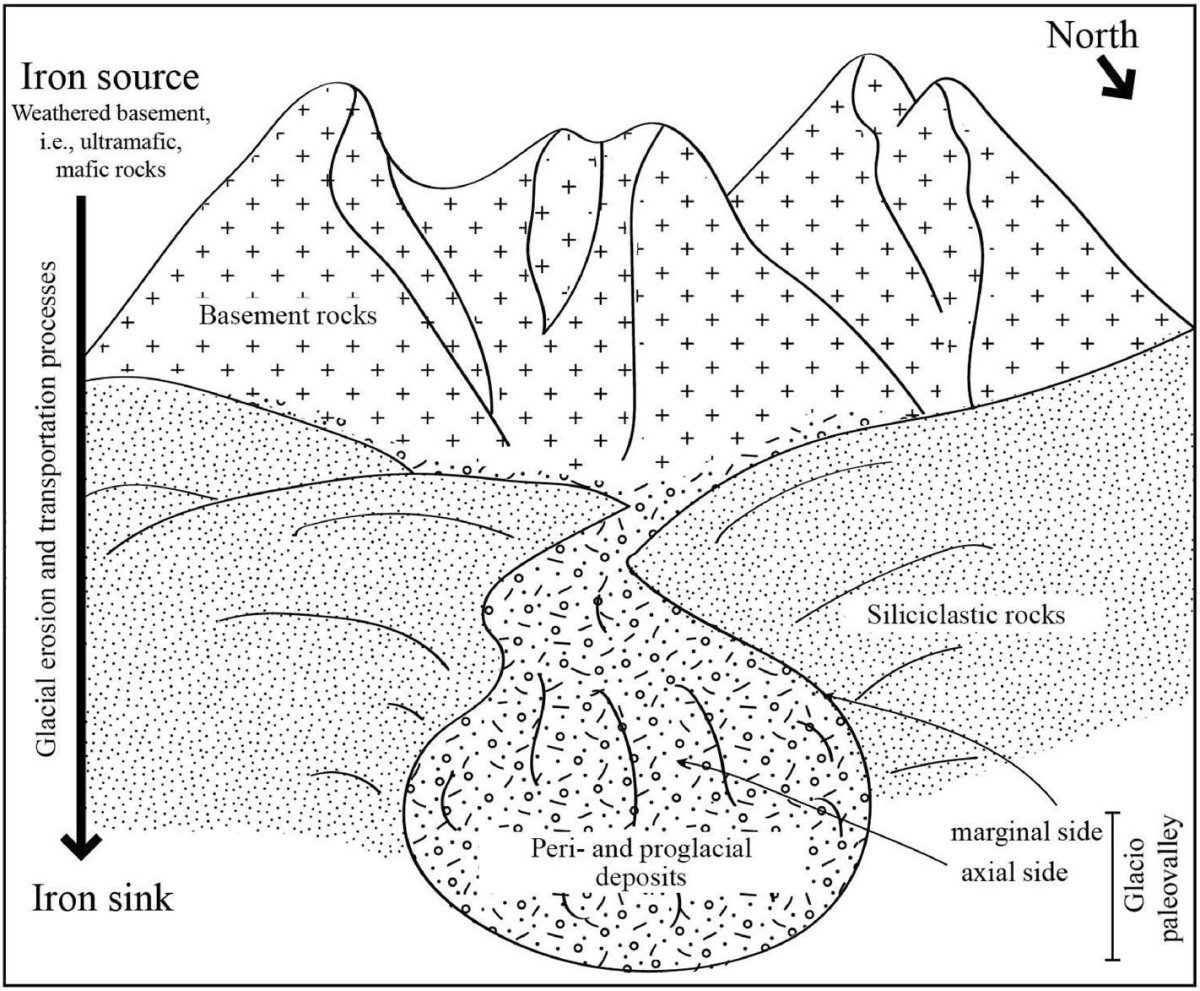
Conceptual depositional model for glacial transportation of iron from the Arabian Shield toward the Arabian basin. Constructed after several published concepts on the Late Ordovician glaciation processes in central Saudi Arabia^32,35,34,37,60^.

The concept of glacial erosion and transportation of basement rocks is evident from ancient and modern glaciation (Fig. 17). It was observed in the same studied deposits of the Late Ordovician glaciogenic deposits that tillites contain basement rocks derived from the Arabian Shield^60,61^. Weathered mafic and ultramafic rocks, laterite, and other basement fragments with higher iron concentrations could be eroded and transported by glacial processes to the studied area and oxidized in a later stage. In modern glaciation, the transportation of iron from the content (the source) to the ocean (the sink) has already been discussed^62^.

The weathered basement rocks of the Arabian Shield are considered the main source of iron in the studied Early Paleozoic sandstones of Saudi Arabia. Glaciation and deglaciation processes are also responsible for redistributing and mechanically transporting the iron content from the weathered basement (the source) to the sandstones of the Early Paleozoic sequences (the sink) (Fig. 17).

### Development of the studied ferruginous deposits

This study reports various ferruginous deposits, including ferruginous spheroidal rinded concretions, pipe, layers, fracture infills, liesegang bands, and ferricrete (Figs. 4, 5, 6, 7, 8). Each type was developed under certain environmental conditions and was controlled by the host rocks' properties, including lithology, grain texture, porosity, and permeability. Under favorable environmental conditions that promote oxidation, iron is oxidized, resulting in the formation of iron-related minerals that are classified as oxides (such as hematite), hydroxides, or oxide-hydroxides (such as goethite)^56^. The development of ferruginous deposits is influenced by a variety of factors, including alterations in the source of iron, tectonic activity, depositional environments, and environmental conditions such as temperature, moisture, pH, and redox state^57,63^.

Most of the studied ferruginous deposits, including layers, fracture infills, liesegang bands, and ferricrete, are expected to develop after the iron in the sediments oxidizes. Before the oxidation, the distribution of iron in the sediments was possibly through the water associated with glacial retreat containing a higher iron concentration, which follows preferential flow paths. The development of such ferruginous deposits was presumably during the final period of glacial retreat, with the absence of vegetation. The deformation in the ferruginous layers observed in S5SA (Fig. 7g,h) might be due to glacial movement or water-escape deformation.

At this stage, it must be admitted that the development of ferruginous concretions is still contentious. Three models were suggested to illustrate the development stages of ferruginous concretions, starting from iron precipitation to deposition. The first model suggested that the iron grain coating was developed diagenetically, removed and mobilized by reducing fluids, and oxidized to develop concretions^2,64,65^. The second model suggested that ferruginous concretions were developed from the dissolution and oxidation of siderite nodules^10,11,20^. The third model suggested that ferruginous concretions were developed after replacing carbonate concretions through chemical processes of pH buffering^21,22^. Chan^65^ has recently discussed these three models and illustrated their strengths and weaknesses. The author suggested that one universal model cannot explain the development of concretions due to a wide range of factors controlling such developments, i.e., scales, age, depositional and post-depositional factors, and other environmental conditions.

### Compare and contrast

**Figure 18:**
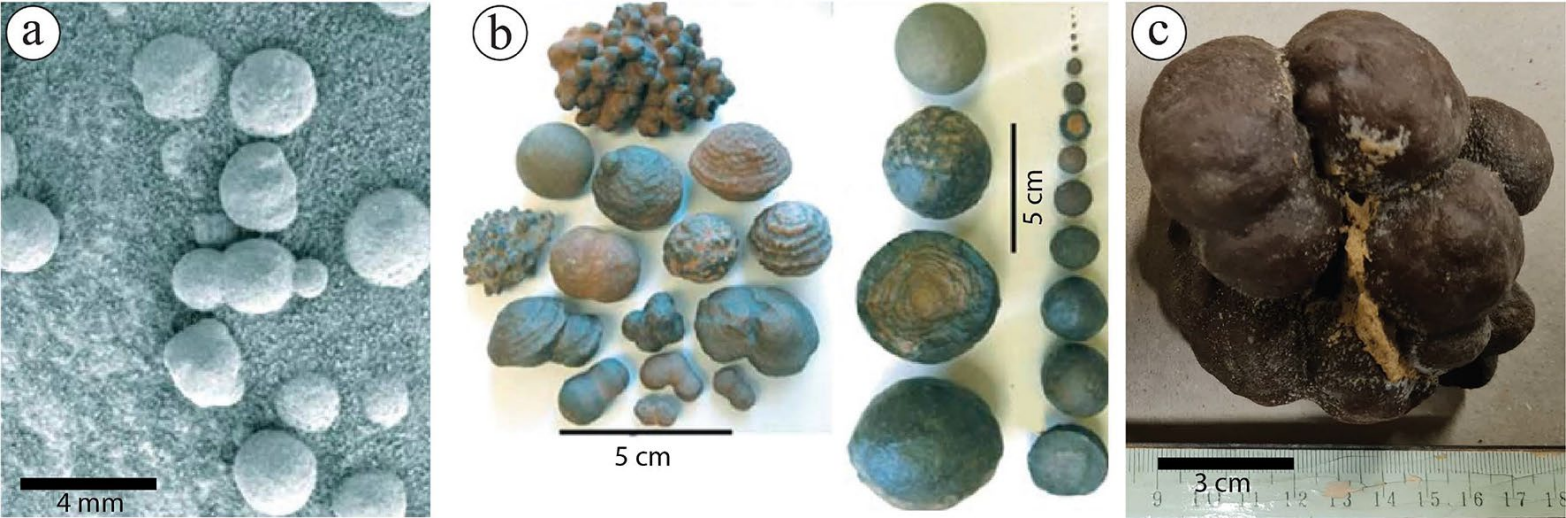
A visual comparison between (**a**) ferruginous concretions of Mars^68^, (**b**) ferruginous concretions reported from the Navajo sandstones in the United States^68^, and (**c**) the ones reported by the current study.

Qualitative comparison has widely been used to understand the characteristics, composition, and distribution of ferruginous deposits worldwide^10,21,65^. Since the discovery of ferruginous deposits, named "Blueberries" (Fig. 18a), in the Burns Formation of the Meridiani Planum on Mars^66,67^, many research works compared such spherules with the ferruginous concretions in the Jurassic Navajo Sandstone^1,2,10,68^ (Fig. 18b). The "Blueberries" are abundant small-sized iron-oxide that are spherules ranging in diameter from 3 to 6 mm^69–72^. The ferruginous concretions of the Navajo sandstone are abundant and vary in size with diameters ranging from millimeters to centimeters and occur as solid, rinded, layered, coalesced, and amalgamated concretions of hematite composition. However, the studied ferruginous concretions can be classified into solid and rinded. The solid ones are low in abundance and found within the glaciofluvial sandstone interval as amalgamated goethite concretions (Fig. 18c). Within the Paleozoic Wajid Sandstones in southwestern Saudi Arabia, Babalola et al.^52^ also reported ferruginous amalgamated concretions but with a higher abundance and of hematite composition. The rinded concretions of the current study were also observed in the glaciofluvial sandstone of the Sarah Formation. They are very large, reaching up to 2 m in their longest diameter, and occur within the sedimentary strata exposed by road cut activities (Fig. 8). El Aal and Nabawy^17^ also reported rinded concretions in the Wajid Sandstone from an area close to Dhahran Al-Janoub, southwestern Saudi Arabia.

**Figure 19:**
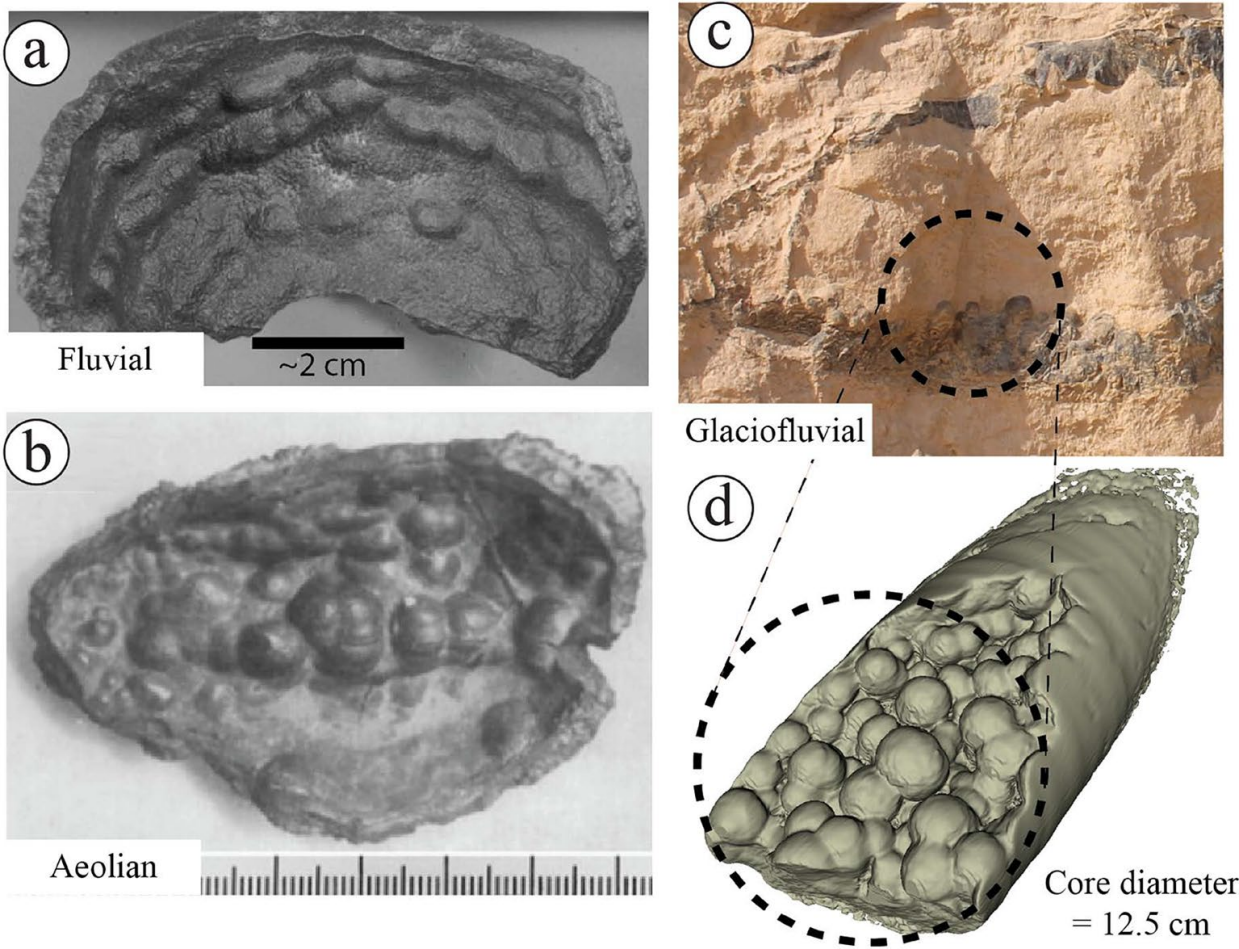
A visual comparison between ferruginous rinded concretions reported from (**a**) the Pleistocene fluvial sediments of the Netherlands^11^, (**b**) the Navajo Sandstone of Utah^10^, and (**c**,**d**) the ferruginous rinded concretion reported by the current study with the aid of CT scans (Supplementary Data).

**Figure 20:**
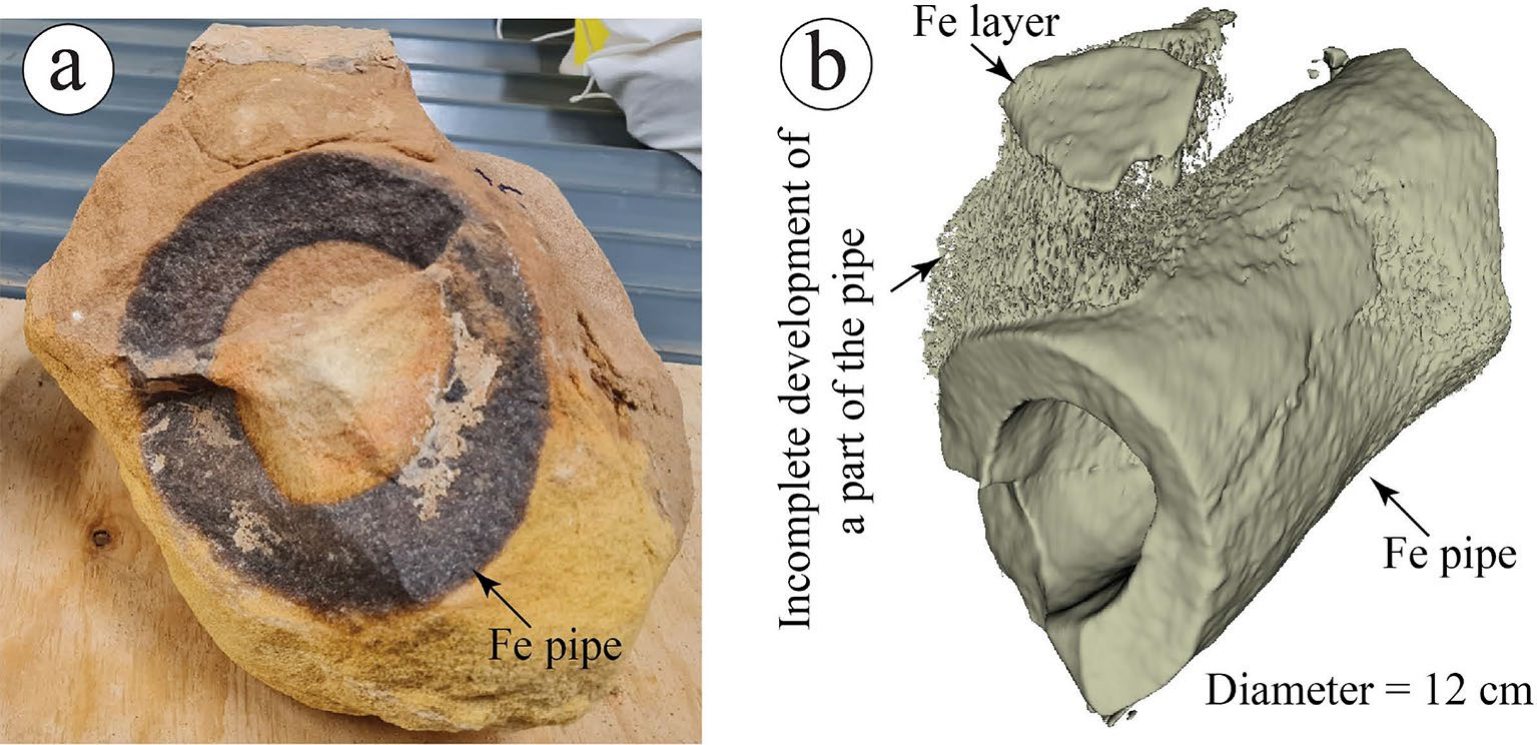
A three-dimensional view of a ferruginous (Fe) pipe (**a**) that can be visualized with the aid of CT scan (**b**, Supplementary Data).

With the help of CT scans, the studied rinded concretions show small concretions on the surface of the rinds, indicating mature internal growth of the concretions (Fig. 19a–d). Despite the major changes in the size and composition, the rinded concretions of the Pleistocene fluvial sediments of the Netherlands and the Navajo Sandstone of Utah^10,11^ did not show well-developed clusters of small concretions on the surface of the rinds as same as the studied concretions. It seems that the depositional and post-depositional controls in the glaciofluvial environment were suitable to develop such concretions better than the fluvial and aeolian settings or might be related to the source of iron in these latter settings. The CT scans of the studied ferruginous pipe show its morphology and how it relates to the ferruginous layer above it (Fig. 20a,b). It appears that the iron got oxidized before the full completion of the development of the pipe, whose length can be more than 1.5 m (Fig. 4f). The ferruginous pipes of the Navajo Sandstone of Utah^1^ and the Shinarump Member of the Chinle Formation, USA^48^ show almost similar morphology, but different composition in comparison with the studied ferruginous pipes.

**Figure 21:**
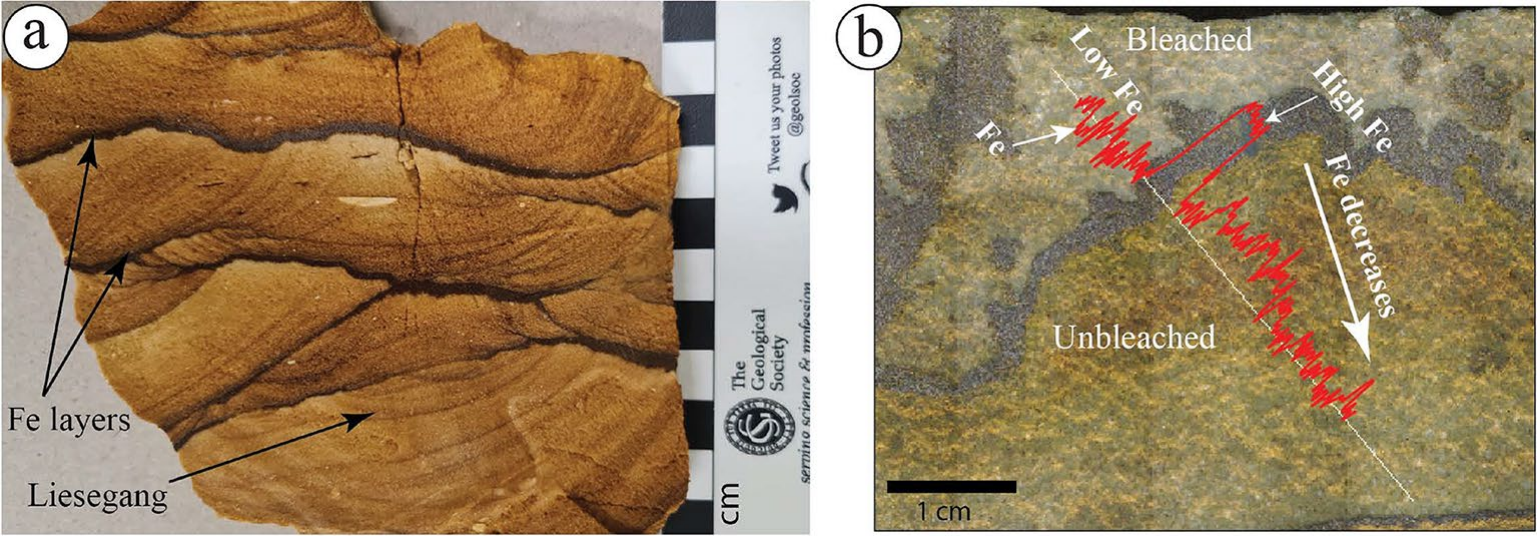
The distribution of (**a**) ferruginous (Fe) layers and liesegang bands, and (**b**) Fe distribution within Fe layers and associated unbleached and bleached areas. Note that Fe layers show a higher concentration of Fe that decreases gradually in the unbleached area and becomes almost absent in the bleached area.

The studied ferruginous layers (dark bands) and liesegang bands (Fig. 21a) show similar patterns with those of Wonderstone of the Shinarump Member, Chinle Formation, USA^48,73^. Kettler et al.^73^ illustrated the differences between the ferruginous layers (dark bands or iron oxide cement) from those of liesegang bands (iron oxide stain). The ferruginous layers are those of iron oxide minerals that occlude pore spaces, while the liesegang bands are those of iron oxide minerals that coat the grains but do not occlude pore spaces^73^. For the Fe distribution between the ferruginous layers and liesegang bands, our µXRF results show that the ferruginous layers are characterized by a relatively very high concentration of Fe (Fig. 21b), which decreases in the stained part of the samples and the liesegang bands (Fig. 10a,b).

It is worth mentioning that quantitative comparison that is beyond the scope of this paper is significant in relating the characteristics of such deposits on regional and global scales. Integrative and comparative studies of the ferruginous deposits provide more insights into the formation mechanism of such deposits. Such investigations would reduce the uncertainty associated with the characteristics, distribution, and scale of investigation in some proposed theories and models for the development of ferruginous deposits on Earth and Mars.

### Applications and significance

There are many applications in life for iron oxides that are of interest to academics, industries, and the environment^18,56,74–76^. Herein, we will only discuss a few aspects that are currently used in sedimentological studies, i.e., the use of ferruginous deposits to infer the geological evolution on Earth and Mars and the impact of ferruginous deposits on the quality of reservoirs and aquifers.

The Earth is being used as an analogue to understand the chemical and mineralogical evolution on the surface of Mars, which is called "the red planet" because of the abundance distribution of iron oxide on its surface, giving it the red color^77,78^. Since the discovery of ferruginous concretions on Mars, which are composed of hematite^69–72^, scientists understand that water is indeed involved in the formation of hematite that was precipitated from water on early Mars^79,80^. It has also been observed from studying the Maritain meteorites that iron oxide minerals, i.e., goethite, can be formed by an aqueous alteration process^79^. Thus, understanding the characteristics, distribution, origin, and development of ferruginous deposits on Earth would provide insights into developing new concepts that would be helpful for geological exploration activities on Mars and other planets.

For the impact of iron oxide distribution on reservoir and aquifer quality, the iron oxide minerals are distributed in the reservoir and aquifers as pore-filling or grain-coating cement. The pore-filling type directly impacts the porosity and permeability by reducing the pore spaces. For instance, the negative impacts of iron oxides on the physical and mechanical properties of the Cambro-Ordovician Wajid Sandstone, the southwestern part of Saudi Arabia^17,81^. Not only that iron oxide cement has a negative impact on reservoir and aquifer quality^82,83^, but also iron oxide with its different mode of occurrences, i.e., pore-filling, coating, or lining, may have an impact on well-logging tools, i.e., resistivity^84^, which leed to miss evaluation of the reservoir and aquifers. Therefore, understanding the distribution of ferruginous content within the sedimentary sequence and its associated minerals would explain features that need to be considered before any evaluation.

## Conclusions

This study illustrated the characteristics, distribution patterns, and origin of various ferruginous deposits in central Saudi Arabia. These ferruginous deposits are observed mainly in the Late Ordovician glaciogenic deposits of the Sarah Formation and also at the topmost part of the shallow marine Quwarah Member of the Qassim Formation and the Sajir Member of the Saq Formation. They are distributed as ferruginous concretions, pipes, undeformed and deformed layers, ferricrete, fracture infills, and unbleached deposits. In the Late Ordovician glaciogenic Sarah Formation, they were more abundant in the coarse-grained sandstones and clast-supported diamictites. The main mineralogical composition of such deposits is goethite, which is expected to be sourced from the weathered basement of the Arabian Shield through glacial erosion and transportation processes. The development of most of the studied ferruginous deposits is controlled by various factors related to the characteristics of the host rocks and other paleoenvironmental conditions. The variations among the suggested models for developing ferruginous concretions reflect that one model is not enough; therefore, more integrative studies from various worldwide locations are needed. Through a comparison between the studied ferruginous deposits and the ones studied on Earth and Mars, this study highlights the significance of further quantitative analysis of representative ferruginous deposits and their associated macro- and micro-features and paleo-depositional settings. Such analysis is expected to improve the models that have been suggested for the development of ferruginous deposits.

### Supplementary Information

Supplementary Information.

## Data Availability

All the data used in this study are available upon request from the corresponding authors.
