# Supplementary figures and images for: Characteristics, distribution, and origin of ferruginous deposits within the Late Ordovician glaciogenic setting of Arabia
Source: Sci Rep. 2023 Oct 27;13:18430. doi: 10.1038/s41598-023-45563-9 (PMC10611803; doi:10.1038/s41598-023-45563-9)

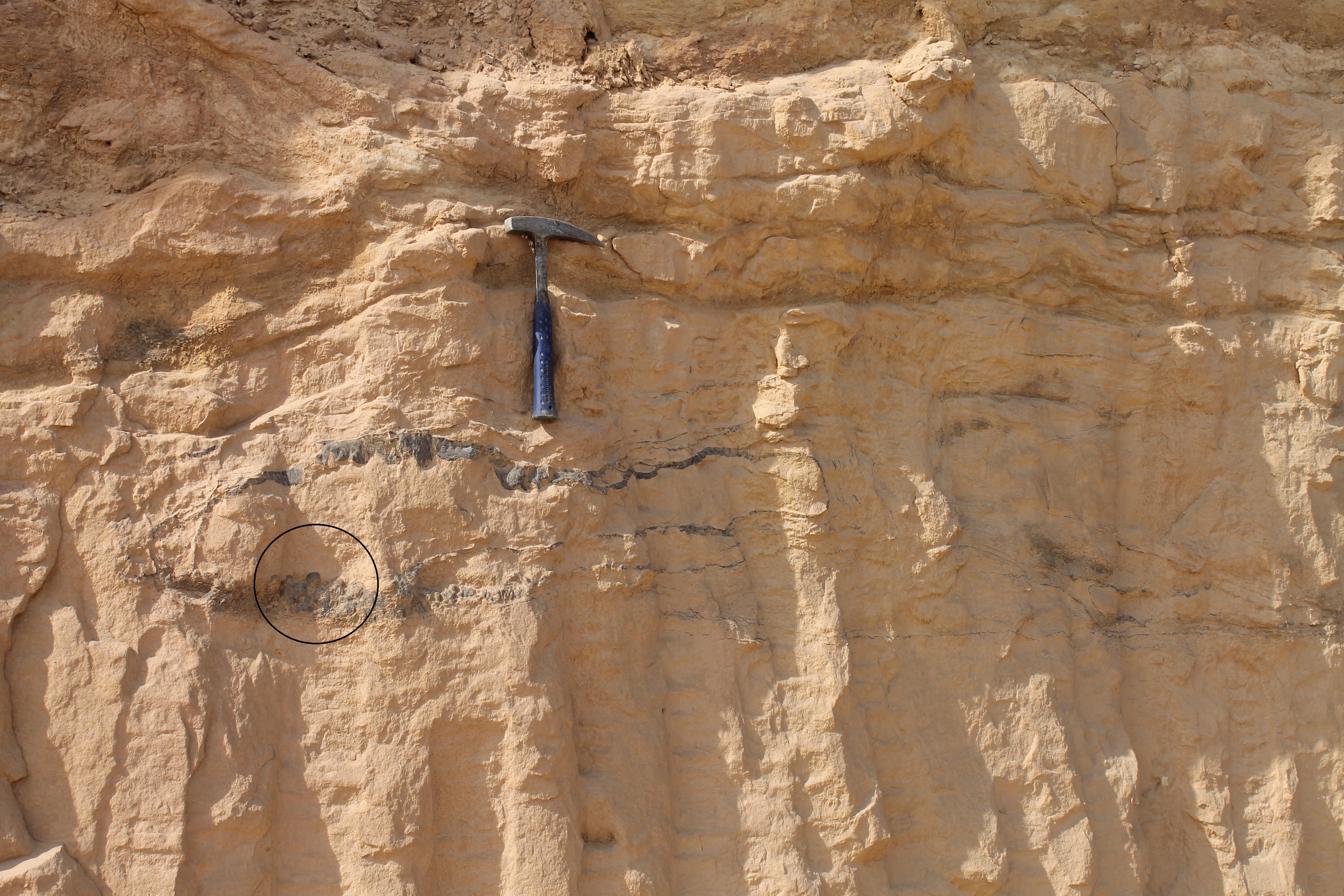

Supplement: Supplementary file 1 — Supplementary Information. [file 41598_2023_45563_MOESM1_ESM.zip › 3D models of Fe deposits/Fe concretions-S6SA-2.1.JPG]

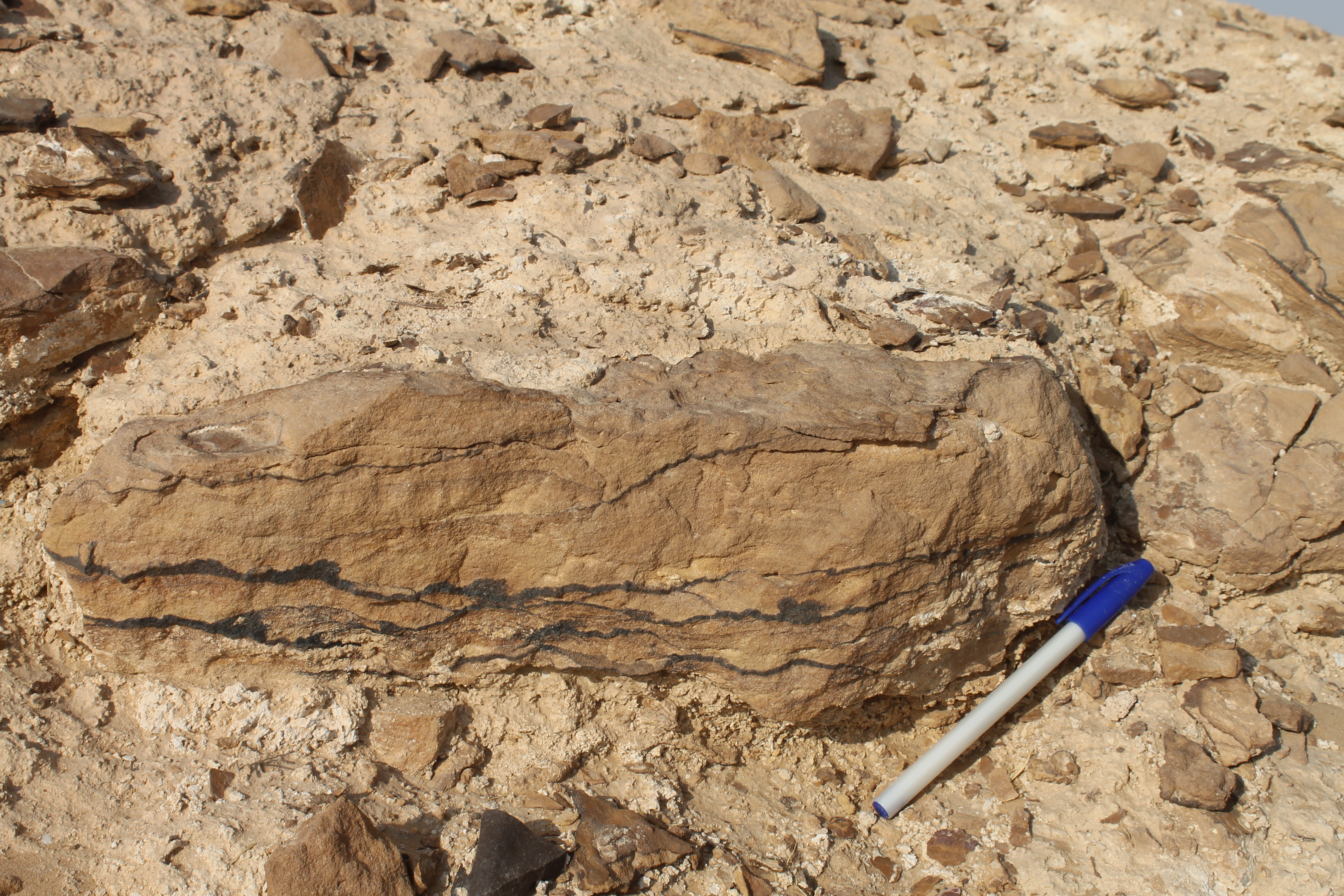

Supplement: Supplementary file 1 — Supplementary Information. [file 41598_2023_45563_MOESM1_ESM.zip › 3D models of Fe deposits/Fe layrs-S1QW-12.JPG]

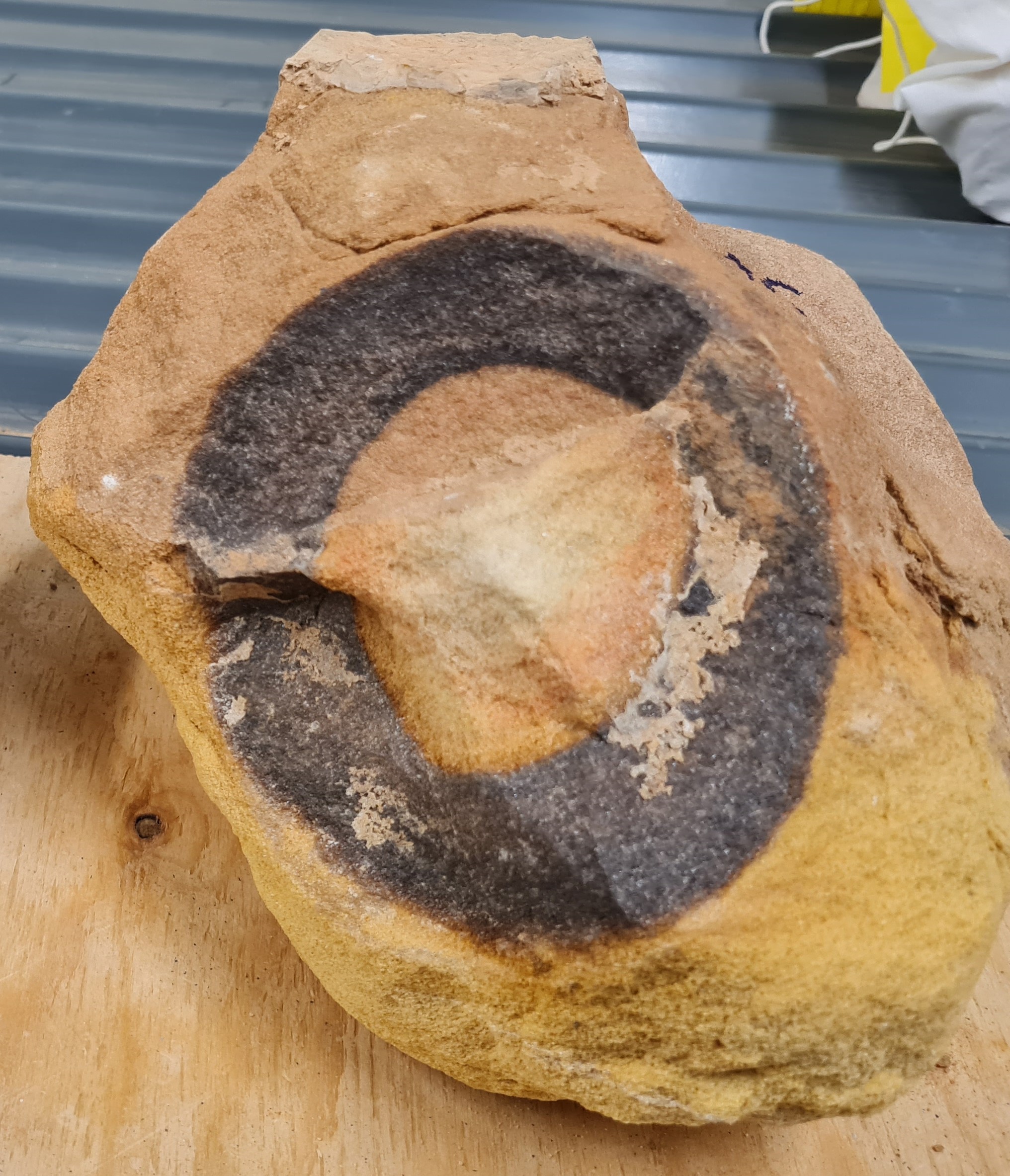

Supplement: Supplementary file 1 — Supplementary Information. [file 41598_2023_45563_MOESM1_ESM.zip › 3D models of Fe deposits/Fe pipe-S4SJ-S10.jpg]
